# Serum Inflammatory Profile for the Discrimination of Clinical Subtypes in Parkinson's Disease

**DOI:** 10.3389/fneur.2018.01123

**Published:** 2018-12-21

**Authors:** Rezzak Yilmaz, Antonio P. Strafella, Alice Bernard, Claudia Schulte, Lieneke van den Heuvel, Nicole Schneiderhan-Marra, Thomas Knorpp, Thomas O. Joos, Frank Leypoldt, Johanna Geritz, Clint Hansen, Sebastian Heinzel, Anja Apel, Thomas Gasser, Anthony E. Lang, Daniela Berg, Walter Maetzler, Connie Marras

**Affiliations:** ^1^Department of Neurology, Christian-Albrechts-University of Kiel, Kiel, Germany; ^2^Institute of Medical Science, University of Toronto, Toronto, ON, Canada; ^3^Edmond J Safra Program in Parkinson's Disease and the Morton and Gloria Shulman Movement Disorders Clinic, University of Toronto, Toronto, ON, Canada; ^4^Division of Neurology, Department of Medicine, University of Toronto, Toronto, ON, Canada; ^5^Research Imaging Centre, Centre for Addiction and Mental Health, Toronto, ON, Canada; ^6^Division of Brain, Imaging and Behaviour-Systems Neuroscience, Toronto Western Research Institute, University Hospital Network, University of Toronto, Toronto, ON, Canada; ^7^Krembil Brain Institute, University Health Network, Toronto, ON, Canada; ^8^Department of Neurodegeneration, Hertie Institute for Clinical Brain Research, University of Tübingen, Tübingen, Germany; ^9^German Center for Neurodegenerative Diseases (DZNE), Tübingen, Germany; ^10^Natural and Medical Sciences Institute (NMI) at the University of Tübingen, Reutlingen, Germany; ^11^Neuroimmunology, Institute of Clinical Chemistry, University Hospital Schleswig-Holstein, Kiel, Germany; ^12^Tanz Centre for Research in Neurodegenerative Diseases, University of Toronto, Toronto, ON, Canada

**Keywords:** Parkinson's inflammation, Parkinson subtypes, immune markers, interleukins, cytokines

## Abstract

**Background:** Blood levels of immune markers have been proposed to discriminate patients with Parkinson's disease (PD) from controls. However, differences between clinical PD subgroups regarding these markers still need to be identified.

**Objective:** To investigate whether clinical phenotypes can be predicted by the assessment of immune marker profiles in the serum of PD patients.

**Methods:** Phenotypes of clinical PD from Tübingen, Germany (*n* = 145) and Toronto, Canada (*n* = 90) were defined regarding clinical subtype, disease onset, severity, and progression as well as presence of cognitive and/or autonomic dysfunction. A panel of serum immune markers was assessed using principal component analysis (PCA) and regression models to define the marker(s) that were associated with clinical phenotypes after adjusting for potential confounders. Findings of both centers were compared for validation. Further, a [18F] FEPPA-PET was performed in a group of patients with high and low values of candidate markers for the assessment of *in vivo* brain microglial activation.

**Results:** Overall, serum immune markers did not cluster to define a pro/anti-inflammatory profile in PCA. Out of 25 markers only IL-12p40 showed a trend to discriminate between PD subgroups in both cohorts which could not be replicated by [18F] FEPPA-PET.

**Conclusions:** Assessment of cytokines in serum does not reliably differentiate clinical PD subtypes. Accompanying subtype-irrelevant inflammation in PD, dual activity, and lack of specificity of the immune markers, the complex function of microglia, probable effects of treatment, disease stage, and progression on inflammation as well as current technical limitations may limit the usefulness of serum immune markers for the differentiation of subtypes.

## Introduction

Parkinson's disease (PD) is one the most frequent movement disorders affecting about 2–3% of the aging population (1). It has been estimated that the number of people with PD will double by 2030, indicating a progressive increase in the socio-economic burden in the near future (1). For this reason, understanding the underlying neurodegeneration is vital and the development of disease modifying therapies is urgently needed.

The pathological process which eventually leads to both a progressive loss of dopaminergic neurons of the substantia nigra and more widespread neurodegeneration in PD has been associated with neuronal inflammation (1, 2). It has been shown that accumulation of extracellular alpha-synuclein (α-syn) facilitates microglia activation, which further promotes production of immune markers, nitric oxide, and reactive oxygen species. It is proposed that this process may lead to nigral cell death. Within this context, pro-inflammatory cytokines produced by the microglia are responsible for the activation of the neighboring inactive glial cells and thus magnify the inflammation. In addition they attach to surface cytokine receptors of the dopaminergic cells and trigger apoptosis (3). This mechanism may provoke protein misfolding and thus create a vicious cycle (1). Evidence for inflammation has been demonstrated in PD animal models (4) as well as in PD patients in genetic (5, 6), imaging (7), CSF (8, 9), and postmortem studies (10, 11).

PD shows considerable variability with regard to age of onset, clinical manifestations, and disease progression which may indicate pathophysiological heterogeneity. The extent that inflammatory processes differ and potentially contribute to clinical PD subtypes has not been elucidated. Detection of a clinical PD phenotype that associates with an inflammatory profile would be critical in terms of outlining the contribution of the immunologic mechanisms to the underlying pathology, understanding the diversity in issues such as drug-response, or disease progression as well as formulating disease modifying strategies. For this reason, we set out to determine the clinical phenotype that correlates with the presence or absence of an inflammatory profile by extensive clinical phenotyping of PD patients and exploratory analysis of inflammatory markers. The results were validated by the assessment of a separate PD cohort and further evaluated by assessing microglial activation *in vivo* using [18F] FEPPA-PET.

## Materials and Methods

The current study has been designed as a collaborative project between the German Center for Neurodegenerative Diseases (DZNE), Tübingen, Germany and the University Health Network, Toronto, Canada which was supported by the Centers of Excellence in Neurodegeneration (CoEN) initiative.

### Study Design

In this cross-sectional study, two separate prospectively assessed cohorts of PD patients from the centers of Tübingen and Toronto were analyzed independently. In each center, patients were grouped according to their clinical phenotypes (see methods below) and then compared with regard to serum inflammatory markers in order to define a clinical PD phenotype associated with inflammation. Then, the findings of both centers were compared for validation. In case of an agreement between two centers regarding the inflammatory markers that differ between clinical phenotypes, [18F] FEPPA-PET imaging was subsequently performed on a subset of patients to further validate the relevance of the inflammatory marker.

The study was approved by the ethics committees of the Medical Faculties of the University of Tübingen and the University Health Network, Toronto. All procedures were in accordance with the Declaration of Helsinki and a written informed consent was obtained from all participants.

### Patients and Clinical Assessments

Participants older than 40 years fulfilling the criteria for idiopathic PD according to the UK Brain Bank Criteria were recruited from the neurology department of University Hospital of Tübingen, Germany, and from the Morton and Gloria Shulman Movement Disorders Clinic of Toronto Western Hospital, Toronto, Canada. Demographic data and detailed PD history including disease onset and duration, medication, presence of wearing off or motor fluctuations, and accompanying non-motor symptoms were collected. Motor performance in the “on” medication state and the disease severity were assessed by the Movement Disorders Society-Unified Parkinson's Disease Rating Scale (MDS-UPDRS) part-III and Hoehn and Yahr Scale (H&Y), respectively. Cognitive performance and autonomic dysfunction were assessed using the Montreal Cognitive Assessment (MoCa) and by Scales for Outcomes in Parkinson's disease—Autonomic (SCOPA-AUT), respectively. Patients with a C-reactive protein (CRP) value above 1.0 mg/dl were excluded from the study based on the assumption that these individuals may have an acute infection that could influence the levels of the collected inflammation markers. All included PD patients were categorized into different groups with regard to their disease onset, severity or progression rate, autonomic, and cognitive status. Based on the previously published cluster analyses defining PD subtypes (12–15), four different methods of subtype classification were constructed. Each method resulted in 2–4 groups with each group representing a clinically defined PD subtype (Table 1). Within these subtypes, “young onset” was defined by an age of onset of <55 or <60 years in method 1 and 2, respectively. Motor subtyping (tremor dominant or non-tremor dominant) of the groups was selected by evaluating the tremor and postural instability and gait disorder (PIGD) scores of MDS-UPDRS part-III. Disease progression was defined by dividing the MDS-UPDRS part-III scores by disease duration. Cognitive or autonomic impairment were defined by Montreal Cognitive Assessment (MoCA) score lower than 26 and Scales for Outcomes in Parkinson's Disease-Autonomic (SCOPA-AUT) equal to or lower than 16, respectively. The details of the group definitions are explained in the Supplementary Table 1.

**Table 1:** Methods of comparison for PD subtypes (n for Tübingen/Toronto cohort).

**Method-1^**13**^**	***n***	**Method-2**	***n***	**Method-3^**15**^**	***n***	**Method-4^**12, 14**^**	***n***
Young onset	31/23	Young onset and slow progression	45/31	Benign motor	37/16	Benign motor	50/35
Non-tremor dominant	44/20	Late onset and fast progression	38/17	Benign motor with cognitive/autonomic impairment	26/22	Benign motor with cognitive impairment	15/14
Tremor dominant	24/19	–		Poor motor with cognitive/autonomic impairment	56/12	Poor motor with cognitive impairment	37/15
Rapid progression	32/8	–		–		–	

### Biomaterial Collection

The following panel of immune markers composed of pro- or anti-inflammatory cytokines as well as neuroprotective trophic factors were assessed from the blood samples of the PD patients: TNF-α, TNF-β, IL-1α, IL-2, IL-3, IL-4, IL-5, IL-7, IL-8, IL-10, IL-12p40, IL-12p70, IL-13, IL-15, IL-16, IL-18, Brain-derived neurotrophic factor (BDNF), Epithelial Neutrophil Activating Peptide (ENA78), granulocyte-macrophage colony-stimulating factor (GM-CSF), lymphotactin, macrophage-derived chemokine (MDC), macrophage inflammatory protein-1β (MIP-1β), monocyte chemotactic protein-1 (MCP-1), stem cell factor (SCF), thrombopoetin (TPO). In both centers, storing and analyses of biomaterial were performed by means of standard operating procedures using the kit components of the multiplexed immunoassay by Myriad RBM, Austin, TX, USA. Blood samples of Toronto were obtained according to the protocol and were sent frozen to Tübingen for the analyses. The Tübingen samples were also frozen before the analysis in order to allow the comparability. Analysis of the serum inflammatory marker levels was performed using the Luminex 100/200 instrument and data were interpreted using the software developed and provided by Myriad RBM. Details of the serum inflammatory marker analysis are given elsewhere (16).

### [18F] FEPPA-PET Imaging

Assessment of the *in vivo* brain microglial activation using a [18F] FEPPA-PET imaging was planned as a further validation step given that immune markers that discriminate PD groups were detected in both cohorts. A subgroup of patients from the Toronto cohort that underwent [18F] FEPPA-PET to assess microglial activation were categorized according to values of the cytokines of interest being lower or higher than the 50th percentile of the entire group undergoing PET imaging. [18F] FEPPA-PET images were preprocessed and region of interests (ROI) were automatically generated using in-house software, ROMI (17). In brief, ROMI fits a standard template of ROIs to an individual proton density (PD)-weighted MR image based on the probability of white matter, gray matter, and cerebrospinal fluid. The individual MR images were then co-registered to each summed [18F] FEPPA-PET image using the normalized mutual information algorithm so that individual refined ROI template can be transferred to the PET image space to generate the time activity curve for each ROI. Our a priori ROIs included cortical as well as subcortical brain regions such as frontal and temporal lobes, cingulate cortex, occipital lobe, insula, cerebellum, thalamus, and striatum. Total distribution volume (V_T_), of the radioligand concentration in the ROI was measured taking into consideration rs6791 polymorphism binding affinity. The effect of age on the tissue volume was taken into account by partial volume effect correction (PVEC) method. PET scans were obtained with a high resolution PET/CT, Siemens-Biograph HiRez XVI (Siemens Molecular Imaging, Knoxville, TN, U.S.A.).

### Statistics

The collected serum inflammatory markers were compared between the groups of each method with an exploratory approach in both cohorts. Statistical analyses were designed in two steps. First, a principal component analysis (PCA) was performed in the Tübingen (main) cohort with a bigger sample size to identify the cytokines that form a pro- or anti-inflammatory immune profile (factor) that could discriminate groups of PD. As the data could not be reduced to factors which convincingly represent the marker profiles (see results), binomial (for Method-2) and multinomial logistic regression analyses (For Methods-1,3, and 4) were performed for each immune marker in the Tübingen and the Toronto cohort in order to explore the predictive effect of an individual immune marker. Subtype was defined as the dependent variable, and the immune marker as the predictor variable with age, sex, disease duration or levodopa equivalent daily dose (LEDD) as covariates which were added to the model according to the group definition. For example, in Method-2, age, and disease duration were not added to the model since the groups were defined according to these variables. Some cytokines were dichotomized at the median value when the data did not fit into the regression model.

As 13 group comparisons were performed for each immune marker (6 group comparisons for method-1, one for 2, and three for method-3 and 4), alpha value was corrected as *p* < 0.05/13 = 0.0038 for each cytokine (18). A trend in the data was defined as *p* < 0.05. The results from both cohorts were compared in order to see whether the effect from the Tübingen cohort (main cohort with a larger sample size) was verified by the second (Toronto) cohort. Inclusion of a second cohort in the study for the confirmation of the results was performed to reduce the risk of type-1 error, and only significant results from both centers were considered definitive. For the [18F] FEPPA-PET two-group analysis an independent samples *t*-test was performed also corrected for multiple comparison. SPSS Statistics 22.0.0 (SPSS Ltd., Chicago IL) was used for statistical analyses.

## Results

A total of 145 PD patients from Tübingen and 90 from Toronto were included in the analysis. 14 patients were excluded due to elevated CRP. Demographic data are shown in Table 2. IL-12p70 and GM-CSF from Toronto, and IL-2 and lymphotactin from both cohorts were excluded since more than 95% of patients had cytokine levels below analytical limit.

**Table 2:** Demographic characteristics of both cohorts.

	**Tübingen (*n* = 145)**	**Toronto (*n* = 90)**
Age [years], mean *(SD)*	67 (9)	62 (9)
Male sex [%]	66	70
Disease duration [years], mean *(SD)*	6.5 (4.0)	7.7 (4.5)
H&Y Stage (0-5), mean	2.3	1.8
LEDD [mg/day], mean *(SD)*	626 (407)	539 (482)
MDS-UPDRS part-III, mean *(SD)*	28.4 (12.5)	21.1 (10.3)
MoCA, mean *(SD)*	26 (3.3)	26.1 (2.6)
SCOPA-AUT, mean *(SD)*	14.3 (7.8)	13.4 (7)

*H&Y, Hoehn & Yahr; LEDD, Levodopa equivalent daily dose, MDS-UPDRS, Movement Disorders Society - Unified Parkinson's Disease Rating Scale; MoCA, Montreal Cognitive Assessment; SCOPA-AUT, Scales for Outcomes in Parkinson's Disease - Autonomic*.

### Principal Component Analysis (PCA)

**Figure 1:**
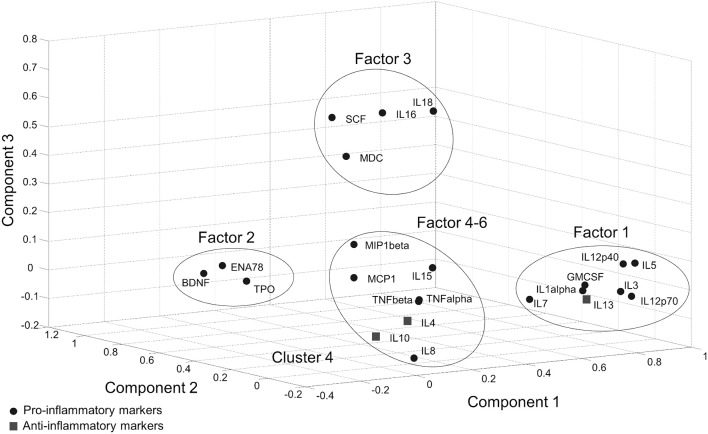
Score plot of the component values of individual inflammatory markers in PCA.

In the Tübingen cohort, a PCA was conducted on serum inflammatory markers with oblique (related) rotation (oblimin) and elimination of factor loadings <0.4. The Kaiser–Meyer–Olkin measure of sampling adequacy was 0.81, verifying the adequacy of the sample size for the analysis. Bartlett's test of sphericity [chi-square(253) = 2125.5, *p* < 0.001] indicated that correlations between items were sufficiently large for PCA. Six components had eigenvalues over Kaiser's criterion of 1 and in combination explained 67% of the variance (Supplementary Table 2). It was found that Factor-1, which has the highest loading (28%), included pro-inflammatory cytokines as well as IL-13 which is an anti-inflammatory cytokine. Likewise, Factor 5 included both pro- (IL-15) and anti-inflammatory (IL-4, IL-10) cytokines. According to the markers that gather on the same factors, a clear separation between pro- and anti-inflammatory marker profiles could not be achieved with PCA (Figure 1, Supplementary Table 2).

### Logistic Regression Analyses

In the Tübingen cohort, IL-1α and IL-3 (at median), and in Toronto cohort IL-1α, IL-12p40 (at median), IL-3 and IL-5 (as >0) were dichotomized to better fit in the model. None of the analyzed cytokines from either cohort reached significance (*p* < 0.0038) with regard to subtype membership prediction except for BDNF in the Toronto cohort (*p* < 0.003), which could not be confirmed in the Tübingen cohort. Some other cytokines showed a tendency (*p* < 0.05) for group separation. In some markers, a significant confounding effect of age or LEDD (not gender and disease duration) was detected which was inconsistent between cohorts. Comparison of the results in both cohorts is presented in Supplementary Table 3.

Out of all cytokines analyzed, only IL-12p40 showed a trend toward significance in both cohorts for distinguishing the “Benign motor” from the “Poor motor with cognitive impairment” group (Method-4, Supplementary Table 3). In the Tübingen cohort, lower values of IL-12p40 were associated with the “Poor motor with cognitive impairment” group against “Benign motor” group independent from the effects of age, sex, disease duration, and LEDD [Nagelkerke *R*^2^ 23%, B = −3.8, *p* = 0.03, Exp(B) = 0.02]. Likewise, the Toronto cohort showed that individuals with a high IL-12p40 value were less likely to be in the “Poor motor with cognitive impairment” group compared to the “Benign motor” group [Nagelkerke *R*^2^ = 35%, B = −2.16, *p* = 0.02, Exp(B) = 0.12] (Supplementary Figure 1). Details of logistic regression results of other cytokines are given in the Supplementary Table 4.

### [18F] FEPPA-PET Imaging

**Figure 2:**
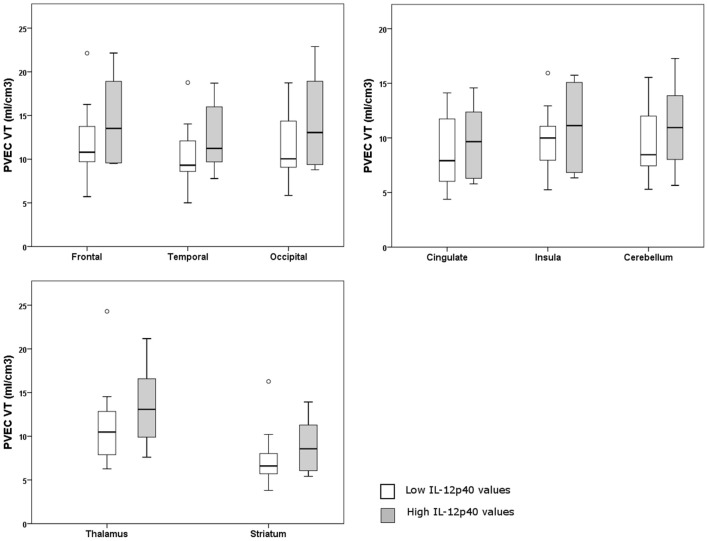
Graphs of partial volume effect corrected (PVEC) total distribution volume (V_T_) in different brain regions in individuals with higher or lower IL-12p40 values.

A [18F] FEPPA-PET imaging from 8 regions of brain was performed for IL12-p40, since it was the only (trend toward an) effect that could be replicated in the second cohort. Eighteen participants from Toronto were divided into two groups according to the lower and upper half of IL-12p40 values. Individuals in both groups showed a mixture of clinical phenotypes. No significant difference was detected between individuals with high or low values of IL-12p40 regarding the microglial activation from different regions of the brain (*p* > 0.10) (Figure 2). Details of the statistical analysis of brain regions are shown in Supplementary Table 5.

## Discussion

In this study, we compared serum immune markers in PD patients with different clinical subtypes in two independent cohorts. The results show that our assessment of serum immune markers, based on the current state of the art of technology, does not sufficiently discriminate clinical subgroups of PD.

Assessing immune marker levels in blood is easily accessible, and therefore has been frequently used to investigate the differences between PD patients and healthy controls. Although there are variations in study design, analysis technology, and findings in these studies (19), peripheral levels of some immune markers seem to be increased in PD patients indicating an ongoing inflammatory process that may trigger or at least accompany neurodegeneration (19). Some of these studies additionally investigated correlations between marker levels and several clinical characteristics such as motor worsening, disease severity, and non-motor status (20–23). Although the results are inconclusive, a further question was raised whether a substantial increase in already existing inflammation may be associated with non-motor features such as autonomic disturbances or dementia. To date, few studies have focused on relationships with non-motor features as the primary outcome. In a 3-year longitudinal study, William-Gray et al. reported that a pro-inflammatory immune marker profile predicted disease progression and correlated with Mini-Mental State Examination scores (24). In another study, Brockmann et al. separated PD patients with LRRK2 mutations according to the presence of non-motor symptoms and found that some pro-inflammatory marker levels were higher in the subgroup with a greater non-motor burden (16).

Contrary to these results, we failed to find an association with these disease specific features. Although some markers showed a trend (*p* < 0.05), no clear finding could be extracted from the results of both cohorts for separating subtypes. On this basis, it can be argued that a direct or linear association between serum marker levels and symptom profile is unlikely in a complex disorder like PD, which is comprised of a variety of motor, autonomic, cognitive, psychiatric, and sensory symptoms from distinct domains with different onset and progression rates. Besides, studies investigating immune markers in blood samples for a relatively regional neurodegeneration of the central nervous system have important limitations. For example, these immune markers are not specific for neuroinflammation and a co-existing systemic inflammation/infection should be taken into account which was considered only in few of the studies (22, 24). Perhaps even a subclinical infection which cannot be easily ruled out could compromise the results. In our study, we have excluded patients with CRP >1.0 mg/dl at least to rule out clinically relevant infections, but we can still not be entirely sure whether the results were biased by an unknown systemic or regional inflammatory process. Therefore, even if an increase in serum immune markers was detected, this increase may not be indicative of neuroinflammation related to the CNS pathogenesis of PD but rather another overlapping comorbidity such as a metabolic syndrome, rheumatic disease, or another accompanying inflammatory process (e.g., via dysphagia and consecutive subtle pulmonary infection, via urinary symptoms with consecutive infection) secondary to PD since these patients are relatively prone to systemic infections (25).

Immune markers may also have both pro- and anti-inflammatory functions according to the stage of inflammation. Accumulating evidence shows that some of the cytokines, even the well-known pro-inflammatory cytokines such as TNF-α, IFN-δ, or IL-6 may have a dual function (26–29), which supports the idea that functional distinction of cytokines as pro- or anti-inflammatory is too simplistic and may not adequately describe the actual inflammatory landscape (30). In our study, we found a trend for IL-12p40 to be decreased in more affected PD patients in both cohorts. This result is in line with recent literature about IL-12p40 in Alzheimer disease (31). Alternatively, although IL-12p40 is primarily a pro-inflammatory marker, it can also stimulate an immunosuppressive response when only slightly increased (32), supporting the dual-function hypothesis. This dual function of the cytokines may have prevented the clear separation of factor loadings in our PCA analysis which was also observed in William-Gray et al. (24). In both studies, the component with a pro-inflammatory profile also included anti-inflammatory cytokines indicating that these immune markers act closely in a complex network and cannot be used to determine a PD subgroup.

The levels of serum immune markers are also affected by a variety of factors such as nutrition (33), body mass index (34), sleep (35), smoking (36), thyroid hormone levels (37), exercise (38, 39), drinking coffee (40, 41), frailty (42), or depression (43, 44), and thus can be considered to be non-specific. These factors could have contributed to the negative outcome of this study. They may also explain the controversial literature that exists for levels of IL-15 (45, 46), IL-6 (47–49), TNF-α (49, 50), IL-10 (32, 51) in association with PD occurrence, TNF-α in association with cognitive scores (20, 52), and IL-6 with UPDRS (24, 53), cognition (20, 54), and depression scores (22, 48) in PD patients. Similarly, results are also conflicting in Alzheimer's disease (55). Considering that all these factors that can hardly be controlled, results from such studies should be interpreted with appropriate caution.

Moreover, intrinsic factors such as treatment and disease progression may contribute to the high variability of immune markers. Little is known about the effect of antiparkinsonian treatment on ongoing inflammation. Several reports have shown that dopamine may have some effect on the immune response (56, 57). Based on these findings, the effect of treatment on immune markers was investigated in some studies (45, 46) or has been taken into account by adding LEDD in the regression model as a confounder (including our study), but not all studies have done this (20, 52). Furthermore, over the course of the progressive disease, the inflammation rate and therefore the blood level of markers may vary since the inflammatory response may modify itself due to the changes in the amount of cells alive, receptor count, or accumulation of α-syn (58). Diversity in the inflammatory response in different brain regions, and up/downregulations in mRNA expression of pro-inflammatory markers between early and late Braak stages have been demonstrated (59). Besides, activation of microglia by α-syn may result in upregulation of both toxic and neuroprotective activity of the microglia (60), further contributing to the limitations in the interpretation of immune markers in PD.

Limitations of the current study have to be mentioned. Eliminating patients with a serum CRP level >1 may have affected our results i.e., may party explain the non-significant results. The serum levels of other positive or negative acute-phase proteins such as procalcitonin, alpha-1 antitrypsin, albumin, or ferritin could also have been alternatively considered for a better detection of individuals with an acute infection. Moreover, we have only considered age, sex, disease duration, and LEDD as potential confounders. Other above mentioned factors such as body mass index, exercise, sleep, smoking, anti-inflammatory drug usage as well as potential comorbidities such as rheumatic or cardiovascular diseases were not taken into account. The possible distortion of results during the transport of frozen blood samples from Toronto and the cross-sectional study-design are also limitations. On the other hand, comparison of the findings in two independent and prospectively assessed cohorts, and although with a small sample, PET imaging are the strengths of the present study. Considering the lack of convincing associations in either cohort, and considering the ambiguous results from previous literature, we conclude that immune mechanisms in PD are far more complex than previously thought. Future longitudinal studies should include strictly designed grouping and confounder assessments to reveal whether specific inflammatory backgrounds are associated with and potentially account for clinical diversity in PD.

## Author Contributions

AS, TG, AL, DB, WM, and CM conceived the research project. RY, AB, AA, TG, AL, SH, DB, WM, and CM organized the research project. AS, AB, CS, LvdH, NS-M, TK, TJ, TG, AL, DB, WM, and CM executed the research project. RY, AB, LvdH, FL, JG, WM, and CM designed the statistical analysis. RY, AS, SH, and AA executed the statistical analysis. RY and CH wrote the first draft in the manuscript preparation. All authors reviewed and critiqued the statistical analysis and manuscript preparation.

### Conflict of Interest Statement

AS received research funding from Canadian Institute of Health Research, Canada Research Chair Program, National Parkinson Foundation, Parkinson Disease Foundation, Parkinson Society Canada, and Weston Brain Institute. TJ serves on the editorial board of Proteomics, Drug Discovery Today. He has received funding for travel or consultancy from Luminex and Myriad RBM. FL reports honoraria by Grifols, Beyer, Roche, Teva, and Biogen, and is an advisory board member for Roche and Biogen. He performs commercial antibody testing in the Institute of Laboratory Medicine, University Hospital Schleswig-Holstein, without personal benefits. TG serves on the editorial boards of *Parkinsonism and Related Disorders, Movement Disorders*, and *Journal of Neurology*; holds a patent re: Kinase Associated with Parkinsonism with Pleiomorphic Pathology (KASPP) (LRRK2) Gene, its Production and Use for the Detection and Treatment of Neurodegenerative Diseases; serves as a consultant for Cephalon, Inc., and Merck Serono; serves on speaker's bureaus of Novartis, Merck Serono, SCHWARZ PHARMA, Boehringer Ingelheim, and Valeant Pharmaceuticals International; and receives research support from Novartis, the European Union, BMBF (the Federal Ministry of Education and Research), and Helmholtz Association. AL has served as an adviser for Abbvie Inc., Allon Therapeutics, Avanir Pharmaceuticals, Biogen Idec, Boerhinger-Ingelheim, Ceregene, Lilly, Medtronic, Merck, Novartis, NeuroPhage Pharmaceuticals, Teva, and UCB; received honoraria from Teva, UCB, AbbVie Inc.; received grants from Brain Canada, Canadian Institutes of Health Research, Edmond J Safra Philanthropic Foundation, Michael J. Fox Foundation, the Ontario Brain Institute, National Parkinson Foundation, Parkinson Society Canada, Tourette Syndrome Association, W. Garfield Weston Foundation; received publishing royalties from Saunders, Wiley-Blackwell, Johns Hopkins Press, and Cambridge University Press; and has served as an expert witness in cases related to the welding industry. DB has served on scientific advisory boards for Novartis, Union chemique belge (UCB)/SCHWARZ PHARMA, Lundbeck, and Teva Pharmaceutical Industries Ltd.; has received funding for travel or speaker honoraria from Boehringer Ingelheim, Lundbeck Inc., Novartis, GlaxoSmithKline, UCB/SCHWARZ PHARMA, Merck Serono, Johnson & Johnson, and Teva Pharmaceutical Industries Ltd.; and has received research support from Janssen, Teva Pharmaceutical Industries Ltd., Solvay Pharmaceuticals, Inc./Abbott, Boehringer, UCB, Michael J Fox Foundation, Bundesministerium für Bildung und Forschung (BMBF), Deutsche Parkinson Vereinigung (dPV) (German Parkinson's disease association), Neuroallianz, DZNE, and Center of Integrative Neurosciences. WM receives or received funding from the European Union, the Michael J. Fox Foundation, Robert Bosch Foundation, Neuroalliance, Lundbeck and Janssen, and holds part of a patent for the assessment of dyskinesias (German patent office, 102015220741.2). He received speaker honoraria from GlaxoSmithKline, Abbvie, UCB, Licher MT and Rölke Pharma, and was invited to Advisory Boards of Market Access & Pricing Strategy GmbH and Abbvie. CM has been consultant for Pfizer, has received honoraria for teaching from EMD Serono, and has received grants from the Michael J Fox Foundation, Canadian Institutes of Health Research, National Parkinson Foundation and the Parkinson Society Canada; Site PI for clinical trial sponsored by Alllon Therapeutics, Physician Services Incorporated, International Parkinson and Movement Disorders Socitety and the Parkinson Disease Foundation. She is employee of the University Health Network and has a contract from Horizon Pharma. The remaining authors declare that the research was conducted in the absence of any commercial or financial relationships that could be construed as a potential conflict of interest.
