# Supplementary material for: Serum Inflammatory Profile for the Discrimination of Clinical Subtypes in Parkinson's Disease
Source: Front Neurol. 2018 Dec 21;9:1123. doi: 10.3389/fneur.2018.01123 (PMC6308160; doi:10.3389/fneur.2018.01123)
Supplement: Supplementary file 1 [file Data_Sheet_1.docx]

**Supplementary File**

Table 1: Subgroup definitions

**Method-1**

| Young onset | Age of onset < 55 years |
| --- | --- |
| Non-tremor dominant | Age of onset ≥ 55 and postural instability and gait disorder (PIGD) dominant phenotype^1^ |
| Tremor dominant | Age of onset ≥ 55 and tremor dominant phenotype^1^ |
| Rapid progressive | MDS-UPDRS part-III*/ Disease duration ≥ 7.5 irrespective of age of onset and tremor |

**Method-2**

| Young onset and slow progression | Age at onset < 60 and MDS-UPDRS part-III/Disease duration ≤ 7. |
| --- | --- |
| Late onset and fast progression | Age at onset ≥ 60 and MDS-UPDRS part-III /Disease duration ≥ 5 |

**Method-3**

| Benign motor | MDS-UPDRS part-III < 26 and MoCA ≥ 26 and SCOPA-AUT ≤ 16 |
| --- | --- |
| Benign motor with cognitive or autonomic impairment | MDS-UPDRS part-III < 26 and MoCA < 26 or SCOPA-AUT > 16 |
| Poor motor with cognitive or autonomic impairment | MDS-UPDRS part-III ≥ 26 and MoCA < 26 or SCOPA-AUT > 16 |

**Method-4**

| Benign motor | MDS-UPDRS part-III < 26 and MoCA ≥ 26 |
| --- | --- |
| Benign motor with cognitive impairment | MDS-UPDRS part-III < 26 and MoCA < 26 |
| Poor motor with cognitive impairment | MDS-UPDRS part-III ≥ 26 and MoCA < 26 |

1 Motor phenotype score:

UPDRS: Tremor score (Mean value of 2.16, 3.20 and 3.21) / PIGD score (mean value of 2.13, 2.14, 2.15, 3.29, 3.30)

PIGD-dominant phenotype: score < 1.0. Tremor dominant phenotype: score > 1.5

MDS-UPDRS: Tremor score (mean value of 2.10, 3.15, 3.16, 3.17 and 3.18) / PIGD score (2.12, 2.13, 3.10, 3.11 and 3.12) PIGD-dominant phenotype: score < 0.90. Tremor dominant phenotype: score > 1.15

* UPDRS part-III was converted into MDS-UPDRS part-III with formula from Goetz et al. [1]

Table 2: Serum inflammatory markers that cluster on factors.

| **Factor 1** (28%) | | **Factor 2** (13%) | | **Factor 3** (8%) | | **Factor 4** (7%) | | **Factor 5** (6%) | | **Factor 6** (5%) | |
| --- | --- | --- | --- | --- | --- | --- | --- | --- | --- | --- | --- |
| IL-12p40 | (0.91) | BDNF | (0.93) | IL-16 | (0.70) | TNF-α | (0.88) | IL-10* | (0.84) | IL-8 | (0.82) |
| IL-3 | (0.89) | TPO | (0.88) | IL-18 | (0.66) | TNF-β | (0.88) | IL-15 | (0.58) | MIP-1β | (0.63) |
| IL-12p70 | (0.87) | ENA78 | (0.54) | SCF | (0.66) |  |  | IL-4* | (0.41) | MCP-1 | (0.55) |
| IL-5 | (0.85) |  |  | MDC | (0.55) |  |  |  |  |  |  |
| IL-1α | (0.81) |  |  |  |  |  |  |  |  |  |  |
| IL-13* | (0.73) |  |  |  |  |  |  |  |  |  |  |
| GM-CSF | (0.71) |  |  |  |  |  |  |  |  |  |  |
| IL-7 | (0.70) |  |  |  |  |  |  |  |  |  |  |

Factor loading of each marker are shown in parenthesis. Loadings < 0.40 were excluded.

% shows explained variance. * Cytokines with anti-inflammatory function.

Figure 1: Box-plot showing the distribution of IL-12p40 values for Method-4 in both cohorts.

**
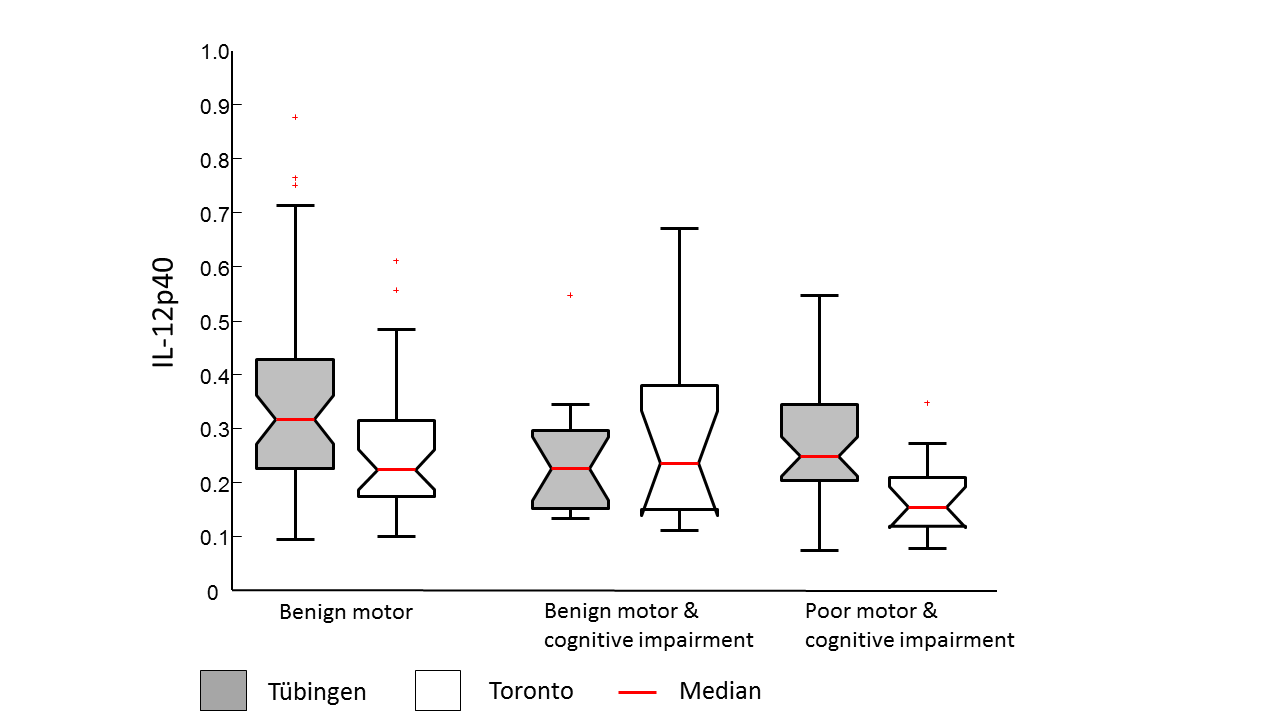
**

Table 3: P values from logistic regression analyses of cytokines for each method from both cohorts.

|  | TNF-α | | TNF-β | | IL-1 α | | IL-3 | | IL-4 | |
| --- | --- | --- | --- | --- | --- | --- | --- | --- | --- | --- |
|  | Tübingen | Toronto | Tübingen | Toronto | Tübingen | Toronto | Tübingen | Toronto | Tübingen | Toronto |
| **Method-1^a^** |  |  |  |  |  |  |  |  |  |  |
| Young onset vs non-tremor dominant | *0.44* | 0.14 | *0.45* | *0.30* | 0.52 | 0.73 | *1.00* | *0.43* | 0.83 | 0.60 |
| Young onset vs tremor dominant | *0.48* | *0.51* | *0.48* | ***0.045*** | 0.16 | 0.97 | 0.75 | 0.81 | 0.24 | *0.69* |
| Young onset vs rapid progression | 0.22 | 0.60 | 0.29 | ***0.03*** | **0.004** | *0.16* | **0.02** | *0.18* | 0.12 | *0.82* |
| Non-tremor dominant vs tremor dominant | *0.91* | ***0.04*** | *0.91* | *0.30* | 0.33 | *0.76* | 0.73 | 0.33 | 0.26 | *0.37* |
| Non-tremor dominant vs rapid progression | **0.045** | 0.49 | 0.07 | *0.12* | **0.008** | *0.09* | **0.008** | *0.45* | 0.12 | *0.50* |
| Tremor dominant vs rapid progression | 0.05 | 0.30 | 0.07 | *0.45* | 0.11 | *0.15* | **0.03** | *0.14* | 0.73 | 0.94 |
|  |  |  |  |  |  |  |  |  |  |  |
| **Method -2^a^** |  |  |  |  |  |  |  |  |  |  |
| Young onset-slow prog. vs late onset-fast prog. | 0.14 | 0.10 | 0.19 | ***0.04*** | **0.04** | *0.38* | 0.14 | *0.41* | 0.29 | *0.38* |
|  |  |  |  |  |  |  |  |  |  |  |
| **Method -3^b^** |  |  |  |  |  |  |  |  |  |  |
| Benign motor vs benign motor-cog/aut. | *0.71* | *0.46* | *0.71* | *0.23* | 0.21 | *0.36* | 0.32 | 0.24 | *0.32* | *0.83* |
| Benign motor vs poor motor-cog/aut. | **0.04** | *0.80* | **0.047** | *0.46* | 0.21 | *0.10* | **0.04** | *0.55* | 0.82 | *0.63* |
| Benign motor-cog/aut. vs poor motor-cog/aut | **0.02** | 0.65 | **0.03** | 0.76 | *0.82* | *0.31* | 0.42 | 0.14 | 0.20 | *0.74* |
|  |  |  |  |  |  |  |  |  |  |  |
| **Method -4^b^** |  |  |  |  |  |  |  |  |  |  |
| Benign motor vs benign motor-cog. | 0.30 | *0.45* | 0.3 | 0.78 | **0.01** | *0.15* | 0.15 | 0.44 | *0.47* | 0.38 |
| Benign motor vs poor motor-cog. | **0.01** | 0.68 | **0.02** | *0.37* | **0.02** | 0.74 | 0.10 | 0.18 | 0.88 | 0.23 |
| Benign motor-cog. vs poor motor-cog. | 0.36 | 0.31 | 0.43 | *0.33* | *0.29* | 0.12 | *0.84* | 0.54 | 0.41 | 0.84 |

a Covariables: sex and LEDD; b covariables: age, sex, disease duration, LEDD

p value of a regression model, in which the increase of the cytokine was associated with the second group, is depicted in italics.

Table 3: P values from logistic regression analyses of cytokines for each method from both cohorts *(continued).*

|  | IL-5 | | IL-7 | | IL-8 | | IL-10 | | IL-12p40 | |
| --- | --- | --- | --- | --- | --- | --- | --- | --- | --- | --- |
|  | Tübingen | Toronto | Tübingen | Toronto | Tübingen | Toronto | Tübingen | Toronto | Tübingen | Toronto |
| **Method-1^a^** |  |  |  |  |  |  |  |  |  |  |
| Young onset vs non-tremor dominant | 0.29 | 0.70 | 0.92 | 0.80 | 0.54 | 0.58 | 0.60 | 0.18 | *0.66* | *0.33* |
| Young onset vs tremor dominant | 0.95 | 0.73 | 0.85 | 0.84 | 0.45 | 0.85 | *0.76* | 0.49 | *0.82* | 0.98 |
| Young onset vs rapid progression | 0.15 | *0.70* | 0.53 | 0.45 | 0.40 | **0.03** | 0.35 | 0.30 | **0.01** | *0.07* |
| Non-tremor dominant vs tremor dominant | *0.35* | *0.96* | 0.90 | *0.95* | 0.54 | *0.71* | *0.41* | *0.47* | 0.90 | 0.32 |
| Non-tremor dominant vs rapid progression | 0.47 | *0.47* | 0.54 | 0.56 | 0.48 | 0.05 | 0.53 | 0.90 | **0.004** | *0.25* |
| Tremor dominant vs rapid progression | 0.16 | *0.51* | 0.65 | 0.53 | 0.94 | **0.03** | 0.26 | 0.57 | **0.006** | *0.07* |
|  |  |  |  |  |  |  |  |  |  |  |
| **Method -2^a^** |  |  |  |  |  |  |  |  |  |  |
| Young onset-slow prog. vs late onset-fast prog. | 0.32 | 1.00 | 0.61 | *0.68* | 0.89 | *0.74* | 0.92 | *0.40* | **0.03** | *0.61* |
|  |  |  |  |  |  |  |  |  |  |  |
| **Method -3^b^** |  |  |  |  |  |  |  |  |  |  |
| Benign motor vs benign motor-cog/aut. | *0.67* | - | 0.30 | 0.97 | 0.57 | 0.29 | *0.16* | *0.50* | 0.54 | *0.39* |
| Benign motor vs poor motor-cog/aut. | 0.08 | 0.81 | 0.41 | *0.78* | 0.38 | 0.47 | *0.22* | *0.42* | 0.06 | *0.26* |
| Benign motor-cog/aut. vs poor motor-cog/aut | **0.04** | - | *0.69* | *0.75* | *0.98* | *0.68* | 0.62 | *0.77* | 0.27 | *0.64* |
|  |  |  |  |  |  |  |  |  |  |  |
| **Method -4** |  |  |  |  |  |  |  |  |  |  |
| Benign motor vs benign motor-cog. | 0.41 | - | 0.13 | 0.75 | 0.71 | 0.09 | *0.09* | *0.53* | **0.03** | *0.86* |
| Benign motor vs poor motor-cog. | 0.06 | *0.44* | 0.43 | **0.04** | 0.52 | 0.33 | *0.32* | 0.42 | **0.03** | **0.02** |
| Benign motor-cog. vs poor motor-cog. | 0.34 | - | *0.33* | 0.12 | *0.91* | *0.43* | 0.23 | 0.22 | *0.36* | **0.03** |

a Covariables: sex and LEDD; b covariables: age, sex, disease duration, LEDD

p value of a regression model, in which the increase of the cytokine was associated with the second group, is depicted in italics.

Table 3: P values from logistic regression analyses of cytokines for each method from both cohorts *(continued).*

|  | IL-12p70 | | IL-13 | | IL-15 | | IL-16 | | IL-18 | |
| --- | --- | --- | --- | --- | --- | --- | --- | --- | --- | --- |
|  | Tübingen | Toronto | Tübingen | Toronto | Tübingen | Toronto | Tübingen | Toronto | Tübingen | Toronto |
| **Method-1^a^** |  |  |  |  |  |  |  |  |  |  |
| Young onset vs non-tremor dominant | 0.82 | - | 0.14 | 0.68 | *0.17* | 0.40 | *0.72* | 0.49 | *0.26* | 0.47 |
| Young onset vs tremor dominant | *0.29* | - | 0.12 | 0.64 | *0.88* | 0.24 | *0.33* | 0.28 | *0.95* | *0.54* |
| Young onset vs rapid progression | 0.14 | - | **0.005** | 0.72 | 0.43 | 0.47 | *0.83* | 0.45 | 0.46 | 0.17 |
| Non-tremor dominant vs tremor dominant | *0.17* | - | 0.64 | 0.15 | 0.30 | 0.75 | *0.45* | 0.67 | 0.35 | *0.21* |
| Non-tremor dominant vs rapid progression | 0.17 | - | 0.06 | 0.96 | **0.04** | 0.91 | *0.91* | 0.78 | 0.07 | 0.33 |
| Tremor dominant vs rapid progression | **0.03** | - | 0.20 | *0.26* | 0.33 | *0.89* | 0.39 | *0.96* | 0.40 | 0.10 |
|  |  |  |  |  |  |  |  |  |  |  |
| **Method-2^a^** |  |  |  |  |  |  |  |  |  |  |
| Young onset-slow prog. vs late onset-fast prog. | 0.34 | - | 0.07 | *0.84* | *0.27* | *0.35* | 0.62 | 0.29 | 0.26 | 0.15 |
|  |  |  |  |  |  |  |  |  |  |  |
| **Method-3^b^** |  |  |  |  |  |  |  |  |  |  |
| Benign motor vs benign motor-cog/aut. | *0.79* | - | 0.38 | 0.86 | *0.08* | *0.36* | 0.48 | *0.96* | 0.06 | 0.53 |
| Benign motor vs poor motor-cog/aut. | 0.18 | - | 0.32 | 0.65 | 0.35 | *0.19* | 0.16 | 0.19 | 0.12 | 0.66 |
| Benign motor-cog/aut. vs poor motor-cog/aut | 0.13 | - | *0.96* | 0.73 | **0.008** | *0.48* | 0.61 | 0.18 | *0.47* | *0.95* |
|  |  |  |  |  |  |  |  |  |  |  |
| **Method-4** |  |  |  |  |  |  |  |  |  |  |
| Benign motor vs benign motor-cog. | 0.18 | - | **0.04** | *0.18* | *0.52* | 0.93 | 0.43 | 0.58 | 0.22 | *0.67* |
| Benign motor vs poor motor-cog. | *0.08* | - | 0.86 | **0.03** | 0.09 | 0.38 | 0.09 | 0.19 | 0.23 | *0.79* |
| Benign motor-cog. vs poor motor-cog. | *0.84* | - | *0.17* | **0.007** | 0.06 | 0.50 | 0.59 | 0.46 | *0.72* | *0.87* |

a Covariables: sex and LEDD; b covariables: age, sex, disease duration, LEDD

p value of a regression model, in which the increase of the cytokine was associated with the second group, is depicted in italics.

Table 3: P values from logistic regression analyses of cytokines for each method from both cohorts *(continued).*

|  | BDNF | | MCP-1 | | MDC | | MIP-1β | | SCF | |
| --- | --- | --- | --- | --- | --- | --- | --- | --- | --- | --- |
|  | Tübingen | Toronto | Tübingen | Toronto | Tübingen | Toronto | Tübingen | Toronto | Tübingen | Toronto |
| **Method-1^a^** |  |  |  |  |  |  |  |  |  |  |
| Young onset vs non-tremor dominant | 0.13 | **0.003** | 0.43 | *0.92* | 0.64 | *0.90* | *0.10* | 0.18 | *0.06* | 0.82 |
| Young onset vs tremor dominant | 0.58 | **0.02** | 0.76 | *0.97* | *0.71* | *0.97* | *0.12* | 0.32 | *0.12* | 0.20 |
| Young onset vs rapid progression | *0.91* | 0.40 | 0.21 | 0.41 | *0.75* | 0.41 | ***0.03*** | 0.70 | *0.67* | 0.68 |
| Non-tremor dominant vs tremor dominant | *0.42* | *0.41* | 0.75 | *0.95* | *0.39* | *0.95* | *0.81* | *0.70* | *0.97* | 0.30 |
| Non-tremor dominant vs rapid progression | *0.11* | *0.09* | 0.49 | 0.36 | *0.41* | 0.36 | *0.29* | *0.49* | 0.18 | 0.80 |
| Tremor dominant vs rapid progression | *0.45* | *0.27* | 0.34 | *0.39* | *0.93* | 0.39 | *0.48* | *0.70* | 0.21 | *0.57* |
|  |  |  |  |  |  |  |  |  |  |  |
| **Method-2^a^** |  |  |  |  |  |  |  |  |  |  |
| Young onset-slow prog. vs late onset-fast prog. | *0.57* | 0.38 | 0.48 | 0.61 | *0.99* | 0.61 | *0.08* | *0.94* | *0.51* | 0.50 |
|  |  |  |  |  |  |  |  |  |  |  |
| **Method-3^b^** |  |  |  |  |  |  |  |  |  |  |
| Benign motor vs benign motor-cog/aut. | 0.79 | 0.55 | 0.29 | 0.98 | *0.39* | *0.98* | **0.02** | 0.11 | *0.09* | 0.37 |
| Benign motor vs poor motor-cog/aut. | 0.38 | 0.59 | 0.06 | 0.84 | *0.48* | *0.84* | 0.41 | 0.87 | *0.98* | 0.83 |
| Benign motor-cog/aut. vs poor motor-cog/aut | 0.61 | 0.91 | 0.71 | 0.81 | 0.77 | *0.81* | *0.07* | *0.23* | 0.05 | *0.54* |
|  |  |  |  |  |  |  |  |  |  |  |
| **Method-4** |  |  |  |  |  |  |  |  |  |  |
| Benign motor vs benign motor-cog. | 0.95 | *0.40* | 0.34 | *0.61* | *0.17* | *0.61* | 0.20 | *0.89* | *0.71* | *0.79* |
| Benign motor vs poor motor-cog. | 0.35 | *0.76* | 0.08 | *0.99* | *0.25* | *0.99* | *0.88* | *0.80* | 0.39 | *0.93* |
| Benign motor-cog. vs poor motor-cog. | 0.52 | 0.63 | 0.68 | 0.66 | 0.62 | 0.66 | *0.18* | *0.74* | 0.31 | 0.76 |

a Covariables: sex and LEDD; b covariables: age, sex, disease duration, LEDD

p value of a regression model, in which the increase of the cytokine was associated with the second group, is depicted in italics.

Table 3: P values from logistic regression analyses of cytokines for each method from both cohorts *(continued).*

|  | TPO | | ENA-78 | | GM-CSF | |
| --- | --- | --- | --- | --- | --- | --- |
|  | Tübingen | Toronto | Tübingen | Toronto | Tübingen | Toronto |
| **Method-1^a^** |  |  |  |  |  |  |
| Young onset vs non-tremor dominant | 0.92 | 0.22 | *0.52* | 0.74 | *0.80* | - |
| Young onset vs tremor dominant | *0.49* | 0.14 | 0.60 | 0.76 | *0.51* | - |
| Young onset vs rapid progression | 0.95 | *0.27* | *0.60* | *0.41* | *-* | - |
| Non-tremor dominant vs tremor dominant | *0.40* | 0.85 | 0.33 | *1.0* | *0.61* | - |
| Non-tremor dominant vs rapid progression | *0.98* | *0.06* | *0.98* | *0.23* | - | - |
| Tremor dominant vs rapid progression | 0.42 | ***0.04*** | *0.34* | *0.27* |  | - |
|  |  |  |  |  |  |  |
| **Method-2^a^** |  |  |  |  |  |  |
| Young onset-slow prog. vs late onset-fast prog. | *0.49* | *0.86* | *0.46* | *0.39* | 0.34 | - |
|  |  |  |  |  |  |  |
| **Method-3^b^** |  |  |  |  |  |  |
| Benign motor vs benign motor-cog/aut. | 0.29 | 0.87 | *0.54* | 0.30 | 0.60 | - |
| Benign motor vs poor motor-cog/aut. | **0.047** | *0.61* | 0.49 | *0.85* | 0.43 | - |
| Benign motor-cog/aut. vs poor motor-cog/aut | 0.47 | *0.49* | 0.18 | *0.27* | 0.94 | - |
|  |  |  |  |  |  |  |
| **Method-4** |  |  |  |  |  |  |
| Benign motor vs benign motor-cog. | 0.36 | *0.80* | *0.17* | *0.94* | - | - |
| Benign motor vs poor motor-cog. | 0.15 | 0.88 | 0.74 | *0.12* | 0.33 | - |
| Benign motor-cog. vs poor motor-cog. | 0.86 | 0.73 | 0.11 | *0.18* | - | - |

a Covariables: sex and LEDD; b covariables: age, sex, disease duration, LEDD

p value of a regression model, in which the increase of the cytokine was associated with the second group, is depicted in italics.

Table 4: Detailed results of logistic regression analyses for Tübingen.

TNF-α:

| Group | B | P value | Exp(B) |
| --- | --- | --- | --- |
| **Method-1 (covariables: sex and LEDD)** |  |  |  |
| Young onset vs non-tremor dominant | 0.07 | 0.44 | 1.00 |
| Young onset vs tremor dominant | 0.08 | 0.48 | 1.08 |
| Young onset vs rapid progression | -0.14 | 0.22 | 0.87 |
| Non-tremor dominant vs tremor dominant | 0.01 | 0.91 | 1.01 |
| Non-tremor dominant vs rapid progression | -0,21 | 0.045 | 0.81 |
| Tremor dominant vs rapid progression | -0,22 | 0.05 | 0.81 |
|  |  |  |  |
| **Method-2 (covariables: sex, LEDD)** |  |  |  |
| Young onset - slow prog. vs late onset - fast prog. | -0.17 | 0.14 | 0.85 |
|  |  |  |  |
| **Method-3 (covariables: age, sex, disease duration, LEDD)** |  |  |  |
| Benign motor vs benign motor - cog/aut. | 0.03 | 0.71 | 1.03 |
| Benign motor vs poor motor - cog/aut. | -0.18 | 0.04 | 0.84 |
| Benign motor - cog/aut. vs poor motor - cog/aut | -0.21 | 0.02 | 0.81 |
|  |  |  |  |
| **Method-4 (covariables: age, sex, disease duration, LEDD)** |  |  |  |
| Benign motor vs benign motor - cog. | -0.12 | 0.30 | 0.89 |
| Benign motor vs poor motor - cog. | -0.23 | 0.01 | 0.80 |
| Benign motor - cog. vs poor motor - cog. | -0.11 | 0.36 | 0.90 |

TNF-β**:**

| Group | B | P value | Exp(B) |
| --- | --- | --- | --- |
| **Method-1 (covariables: sex and LEDD)** |  |  |  |
| Young onset vs non-tremor dominant | 0.07 | 0.45 | 1.28 |
| Young onset vs tremor dominant | 0.08 | 0.48 | 1.08 |
| Young onset vs rapid progression | -0.12 | 0.29 | 0.89 |
| Non-tremor dominant vs tremor dominant | 0.01 | 0.91 | 1.01 |
| Non-tremor dominant vs rapid progression | -0.18 | 0.07 | 0.83 |
| Tremor dominant vs rapid progression | -0.20 | 0.07 | 0.82 |
|  |  |  |  |
| **Method-2 (covariables: sex, LEDD)** |  |  |  |
| Young onset - slow prog. vs late onset - fast prog. | -0.14 | 0.19 | 0.87 |
|  |  |  |  |
| **Method-3 (covariables: age, sex, disease duration, LEDD)** |  |  |  |
| Benign motor vs benign motor - cog/aut. | 0.03 | 0.71 | 1.03 |
| Benign motor vs poor motor - cog/aut. | -0.17 | **0.047** | 0.85 |
| Benign motor - cog/aut. vs poor motor - cog/aut | -0.20 | **0.03** | 0.82 |
|  |  |  |  |
| **Method-4 (covariables: age, sex, disease duration, LEDD)** |  |  |  |
| Benign motor vs benign motor - cog. | -0.12 | 0.30 | 0.89 |
| Benign motor vs poor motor - cog. | -0,21 | **0.02** | 0.81 |
| Benign motor - cog. vs poor motor - cog. | -0.09 | 0.43 | 0.91 |

IL-1alpha:

| Group | B | P value | Exp(B) |
| --- | --- | --- | --- |
| **Method-1 (covariables: sex and LEDD)** |  |  |  |
| Young onset vs non-tremor dominant | -0.31 | 0.52 | 0.73 |
| Young onset vs tremor dominant | -0.85 | 0.16 | 0.43 |
| Young onset vs rapid progression | -1.80 | **0.004** | 0.17 |
| Non-tremor dominant vs tremor dominant | -0.54 | 0.33 | 0.58 |
| Non-tremor dominant vs rapid progression | -1.47 | **0.008** | 0.23 |
| Tremor dominant vs rapid progression | -0.94 | 0.11 | 0.39 |
|  |  |  |  |
| **Method-2 (covariables: sex, LEDD)** |  |  |  |
| Young onset - slow prog. vs late onset - fast prog. | -1.14 | **0.04** | 0.32 |
|  |  |  |  |
| **Method-3 (covariables: age, sex, disease duration, LEDD)** |  |  |  |
| Benign motor vs benign motor - cog/aut. | -0.68 | 0.21 | 0.51 |
| Benign motor vs poor motor - cog/aut. | -0.57 | 0.21 | 0.57 |
| Benign motor - cog/aut. vs poor motor - cog/aut | 0.11 | 0.82 | 1.11 |
|  |  |  |  |
| **Method-4 (covariables: age, sex, disease duration, LEDD)** |  |  |  |
| Benign motor vs benign motor - cog. | -1.92 | **0.01** | 0.15 |
| Benign motor vs poor motor - cog. | -1.13 | **0.02** | 0.32 |
| Benign motor - cog. vs poor motor - cog. | 0.78 | 0.29 | 2.19 |

IL-3:

| Group | B | P value | Exp(B) |
| --- | --- | --- | --- |
| **Method-1 (covariables: sex and LEDD)** |  |  |  |
| Young onset vs non-tremor dominant | 0.002 | 1.00 | 1.00 |
| Young onset vs tremor dominant | -0.19 | 0.75 | 0.83 |
| Young onset vs rapid progression | -1.47 | **0.02** | 0.23 |
| Non-tremor dominant vs tremor dominant | -0.19 | 0.73 | 0.82 |
| Non-tremor dominant vs rapid progression | -1.47 | **0.008** | 0.23 |
| Tremor dominant vs rapid progression | -1.28 | **0.03** | 0.28 |
|  |  |  |  |
| **Method-2 (covariables: sex, LEDD)** |  |  |  |
| Young onset - slow prog. vs late onset - fast prog. | -0.79 | 0.15 | 0.45 |
|  |  |  |  |
| **Method-3 (covariables: age, sex, disease duration, LEDD)** |  |  |  |
| Benign motor vs benign motor - cog/aut. | -0.53 | 0.32 | 0.59 |
| Benign motor vs poor motor - cog/aut. | -0.92 | 0.04 | 0.40 |
| Benign motor - cog/aut. vs poor motor - cog/aut | -0.40 | 0.42 | 0.67 |
|  |  |  |  |
| **Method-4 (covariables: age, sex, disease duration, LEDD)** |  |  |  |
| Benign motor vs benign motor - cog. | -0.91 | 0.15 | 0.41 |
| Benign motor vs poor motor - cog. | -0.78 | 0.10 | 0.46 |
| Benign motor - cog. vs poor motor - cog. | 0.13 | 0.84 | 1.14 |

IL-4:

| Group | B | P value | Exp(B) |
| --- | --- | --- | --- |
| **Method-1 (covariables: sex and LEDD)** |  |  |  |
| Young onset vs non-tremor dominant | -0.007 | 0.83 | 1.00 |
| Young onset vs tremor dominant | -0.05 | 0.24 | 0.95 |
| Young onset vs rapid progression | -0.06 | 0.12 | 0.94 |
| Non-tremor dominant vs tremor dominant | -0.04 | 0.26 | 0.96 |
| Non-tremor dominant vs rapid progression | -0.06 | 0.12 | 0.95 |
| Tremor dominant vs rapid progression | -0.01 | 0.73 | 0.98 |
|  |  |  |  |
| **Method-2 (covariables: sex, LEDD)** |  |  |  |
| Young onset - slow prog. vs late onset - fast prog. | -0.04 | 0.29 | 0.96 |
|  |  |  |  |
| **Method-3 (covariables: age, sex, disease duration, LEDD)** |  |  |  |
| Benign motor vs benign motor - cog/aut. | 0.04 | 0.32 | 1.04 |
| Benign motor vs poor motor - cog/aut. | -0.007 | 0.82 | 0.99 |
| Benign motor - cog/aut. vs poor motor - cog/aut | -0.05 | 0.20 | 0.96 |
|  |  |  |  |
| **Method-4 (covariables: age, sex, disease duration, LEDD)** |  |  |  |
| Benign motor vs benign motor - cog. | 0.03 | 0.47 | 1.03 |
| Benign motor vs poor motor - cog. | -0.01 | 0.88 | 1.00 |
| Benign motor - cog. vs poor motor - cog. | -0.04 | 0.41 | 0.96 |

IL-5:

| Group | B | P value | Exp(B) |
| --- | --- | --- | --- |
| **Method-1 (covariables: sex and LEDD)** |  |  |  |
| Young onset vs non-tremor dominant | -0.12 | 0.29 | 0.88 |
| Young onset vs tremor dominant | -0.007 | 0.95 | 0.99 |
| Young onset vs rapid progression | -0.26 | 0.15 | 0.77 |
| Non-tremor dominant vs tremor dominant | 0.12 | 0.35 | 1.12 |
| Non-tremor dominant vs rapid progression | -0.13 | 0.47 | 0.88 |
| Tremor dominant vs rapid progression | -0.25 | 0.16 | 0.78 |
|  |  |  |  |
| **Method-2 (covariables: sex, LEDD)** |  |  |  |
| Young onset - slow prog. vs late onset - fast prog. | -0.15 | 0.32 | 0.86 |
|  |  |  |  |
| **Method-3 (covariables: age, sex, disease duration, LEDD)** |  |  |  |
| Benign motor vs benign motor - cog/aut. | 0.04 | 0.67 | 1.04 |
| Benign motor vs poor motor - cog/aut. | -0.25 | 0.08 | 0.78 |
| Benign motor - cog/aut. vs poor motor - cog/aut | -0.30 | **0.04** | 0.74 |
|  |  |  |  |
| **Method-4 (covariables: age, sex, disease duration, LEDD)** |  |  |  |
| Benign motor vs benign motor - cog. | -0.16 | 0.41 | 0.85 |
| Benign motor vs poor motor - cog. | -0.42 | 0.06 | 0.66 |
| Benign motor - cog. vs poor motor - cog. | -0.26 | 0.34 | 0.77 |

IL-7:

| Group | B | P value | Exp(B) |
| --- | --- | --- | --- |
| **Method-1 (covariables: sex and LEDD)** |  |  |  |
| Young onset vs non-tremor dominant | -0.002 | 0.92 | 1.00 |
| Young onset vs tremor dominant | -0.005 | 0.85 | 1.00 |
| Young onset vs rapid progression | -0.02 | 0.53 | 0.98 |
| Non-tremor dominant vs tremor dominant | -0.003 | 0.90 | 1.00 |
| Non-tremor dominant vs rapid progression | -0.01 | 0.54 | 1.00 |
| Tremor dominant vs rapid progression | -0.01 | 0.65 | 1.00 |
|  |  |  |  |
| **Method-2 (covariables: sex, LEDD)** |  |  |  |
| Young onset - slow prog. vs late onset - fast prog. | -0.02 | 0.61 | 0.43 |
|  |  |  |  |
| **Method-3 (covariables: age, sex, disease duration, LEDD)** |  |  |  |
| Benign motor vs benign motor - cog/aut. | -0.03 | 0.30 | 0.97 |
| Benign motor vs poor motor - cog/aut. | -0.02 | 0.41 | 0.98 |
| Benign motor - cog/aut. vs poor motor - cog/aut | 0.01 | 0.69 | 1.01 |
|  |  |  |  |
| **Method-4 (covariables: age, sex, disease duration, LEDD)** |  |  |  |
| Benign motor vs benign motor - cog. | -0.05 | 0.13 | 0.95 |
| Benign motor vs poor motor - cog. | -0.02 | 0.43 | 0.98 |
| Benign motor - cog. vs poor motor - cog. | 0.03 | 0.33 | 1.03 |

IL-8:

| Group | B | P value | Exp(B) |
| --- | --- | --- | --- |
| **Method-1 (covariables: sex and LEDD)** |  |  |  |
| Young onset vs non-tremor dominant | -0.002 | 0.54 | 1.00 |
| Young onset vs tremor dominant | -0.009 | 0.45 | 0.99 |
| Young onset vs rapid progression | -0.01 | 0.40 | 0.99 |
| Non-tremor dominant vs tremor dominant | -0.007 | 0.54 | 0.99 |
| Non-tremor dominant vs rapid progression | -0.008 | 0.48 | 0.99 |
| Tremor dominant vs rapid progression | -0.001 | 0.94 | 1.00 |
|  |  |  |  |
| **Method-2 (covariables: sex, LEDD)** |  |  |  |
| Young onset - slow prog. vs late onset - fast prog. | -0.001 | 0.89 | 1.00 |
|  |  |  |  |
| **Method-3 (covariables: age, sex, disease duration, LEDD)** |  |  |  |
| Benign motor vs benign motor - cog/aut. | -0.01 | 0.57 | 0.99 |
| Benign motor vs poor motor - cog/aut. | -0.01 | 0.38 | 0.99 |
| Benign motor - cog/aut. vs poor motor - cog/aut | 0.00 | 0.98 | 1.00 |
|  |  |  |  |
| **Method-4 (covariables: age, sex, disease duration, LEDD)** |  |  |  |
| Benign motor vs benign motor - cog. | -0.01 | 0.71 | 0.99 |
| Benign motor vs poor motor - cog. | -0.01 | 0.52 | 0.99 |
| Benign motor - cog. vs poor motor - cog. | 0.004 | 0.91 | 1.00 |

IL-10:

| Group | B | P value | Exp(B) |
| --- | --- | --- | --- |
| **Method-1 (covariables: sex and LEDD)** |  |  |  |
| Young onset vs non-tremor dominant | -0.03 | 0.60 | 0.97 |
| Young onset vs tremor dominant | 0.02 | 0.76 | 1.02 |
| Young onset vs rapid progression | -0.10 | 0.35 | 0.91 |
| Non-tremor dominant vs tremor dominant | 0.05 | 0.41 | 1.05 |
| Non-tremor dominant vs rapid progression | -0.07 | 0.53 | 0.94 |
| Tremor dominant vs rapid progression | -0.14 | 0.26 | 0.89 |
|  |  |  |  |
| **Method-2 (covariables: sex, LEDD)** |  |  |  |
| Young onset - slow prog. vs late onset - fast prog. | -0.004 | 0.92 | 1.00 |
|  |  |  |  |
| **Method-3 (covariables: age, sex, disease duration, LEDD)** |  |  |  |
| Benign motor vs benign motor - cog/aut. | 0.15 | 0.16 | 1.16 |
| Benign motor vs poor motor - cog/aut. | 0.13 | 0.22 | 1.14 |
| Benign motor - cog/aut. vs poor motor - cog/aut | -0.22 | 0.62 | 0.98 |
|  |  |  |  |
| **Method-4 (covariables: age, sex, disease duration, LEDD)** |  |  |  |
| Benign motor vs benign motor - cog. | 0.22 | 0.09 | 1.25 |
| Benign motor vs poor motor - cog. | 0.11 | 0.32 | 1.12 |
| Benign motor - cog. vs poor motor - cog. | -0.11 | 0.23 | 0.90 |

IL-12p40:

| Group | B | P value | Exp(B) |
| --- | --- | --- | --- |
| **Method-1 (covariables: sex and LEDD)** |  |  |  |
| Young onset vs non-tremor dominant | 0.72 | 0.66 | 2.05 |
| Young onset vs tremor dominant | 0.49 | 0.82 | 1.62 |
| Young onset vs rapid progression | -6.84 | **0.01** | 0.001 |
| Non-tremor dominant vs tremor dominant | -0.24 | 0.90 | 0.79 |
| Non-tremor dominant vs rapid progression | -7.56 | **0.004** | 0.001 |
| Tremor dominant vs rapid progression | -7.33 | **0.006** | 0.001 |
|  |  |  |  |
| **Method-2 (covariables: sex, LEDD)** |  |  |  |
| Young onset - slow prog. vs late onset - fast prog. | -5,166 | **0.03** | 0.006 |
|  |  |  |  |
| **Method-3 (covariables: age, sex, disease duration, LEDD)** |  |  |  |
| Benign motor vs benign motor - cog/aut. | -0,997 | 0.54 | 0.37 |
| Benign motor vs poor motor - cog/aut. | -2,823 | 0.06 | 0.06 |
| Benign motor - cog/aut. vs poor motor - cog/aut | -1,826 | 0.27 | 0.16 |
|  |  |  |  |
| **Method-4 (covariables: age, sex, disease duration, LEDD)** |  |  |  |
| Benign motor vs benign motor - cog. | -6,654 | **0.03** | 0.001 |
| Benign motor vs poor motor - cog. | -3,79 | **0.03** | 0.02 |
| Benign motor - cog. vs poor motor - cog. | 2,864 | 0.36 | 17.54 |

IL-12p70:

| Group | B | P value | Exp(B) |
| --- | --- | --- | --- |
| **Method-1 (covariables: sex and LEDD)** |  |  |  |
| Young onset vs non-tremor dominant | -0.005 | 0.82 | 1.00 |
| Young onset vs tremor dominant | 0.02 | 0.29 | 1.02 |
| Young onset vs rapid progression | -0.06 | 0.14 | 0.95 |
| Non-tremor dominant vs tremor dominant | 0.03 | 0.17 | 1.03 |
| Non-tremor dominant vs rapid progression | -0.05 | 0.17 | 0.95 |
| Tremor dominant vs rapid progression | -0.08 | **0.03** | 0.93 |
|  |  |  |  |
| **Method-2 (covariables: sex, LEDD)** |  |  |  |
| Young onset - slow prog. vs late onset - fast prog. | -0.03 | 0.34 | 0.98 |
|  |  |  |  |
| **Method-3 (covariables: age, sex, disease duration, LEDD)** |  |  |  |
| Benign motor vs benign motor - cog/aut. | 0.005 | 0.79 | 1.01 |
| Benign motor vs poor motor - cog/aut. | -0.03 | 0.18 | 0.97 |
| Benign motor - cog/aut. vs poor motor - cog/aut | -0.03 | 0.13 | 0.97 |
|  |  |  |  |
| **Method-4 (covariables: age, sex, disease duration, LEDD)** |  |  |  |
| Benign motor vs benign motor - cog. | -0.05 | 0.18 | 0.95 |
| Benign motor vs poor motor - cog. | -0.04 | 0.08 | 0.96 |
| Benign motor - cog. vs poor motor - cog. | 0.008 | 0.84 | 1.01 |

IL-13:

| Group | B | P value | Exp(B) |
| --- | --- | --- | --- |
| **Method-1 (covariables: sex and LEDD)** |  |  |  |
| Young onset vs non-tremor dominant | -0.11 | 0.14 | 0.89 |
| Young onset vs tremor dominant | -0.16 | 0.12 | 0.86 |
| Young onset vs rapid progression | -0.28 | **0.005** | 0.76 |
| Non-tremor dominant vs tremor dominant | -0.04 | 0.64 | 0.96 |
| Non-tremor dominant vs rapid progression | -0.17 | 0.06 | 0.85 |
| Tremor dominant vs rapid progression | -0.12 | 0.20 | 0.88 |
|  |  |  |  |
| **Method-2 (covariables: sex, LEDD)** |  |  |  |
| Young onset - slow prog. vs late onset - fast prog. | -0.18 | 0.07 | 0.83 |
|  |  |  |  |
| **Method-3 (covariables: age, sex, disease duration, LEDD)** |  |  |  |
| Benign motor vs benign motor - cog/aut. | -0.07 | 0.38 | 0.93 |
| Benign motor vs poor motor - cog/aut. | -0.07 | 0.32 | 0.93 |
| Benign motor - cog/aut. vs poor motor - cog/aut | 0.004 | 0.96 | 1.004 |
|  |  |  |  |
| **Method-4 (covariables: age, sex, disease duration, LEDD)** |  |  |  |
| Benign motor vs benign motor - cog. | -0.25 | **0.04** | 0.78 |
| Benign motor vs poor motor - cog. | -0.09 | 0.86 | 1.09 |
| Benign motor - cog. vs poor motor - cog. | 0.16 | 0.17 | 1.18 |

IL-15:

| Group | B | P value | Exp(B) |
| --- | --- | --- | --- |
| **Method-1 (covariables: sex and LEDD)** |  |  |  |
| Young onset vs non-tremor dominant | 2,87 | 0.17 | 17,62 |
| Young onset vs tremor dominant | 0.41 | 0.88 | 1.51 |
| Young onset vs rapid progression | -2.04 | 0.43 | 0.13 |
| Non-tremor dominant vs tremor dominant | -2.46 | 0.30 | 0.09 |
| Non-tremor dominant vs rapid progression | -4.91 | 0.04 | 0.007 |
| Tremor dominant vs rapid progression | -2.45 | 0.33 | 0.09 |
|  |  |  |  |
| **Method-2 (covariables: sex, LEDD)** |  |  |  |
| Young onset - slow prog. vs late onset - fast prog. | 2.45 | 0.27 | 11.62 |
|  |  |  |  |
| **Method-3 (covariables: age, sex, disease duration, LEDD)** |  |  |  |
| Benign motor vs benign motor - cog/aut. | 4.00 | 0.08 | 54.33 |
| Benign motor vs poor motor - cog/aut. | -1.08 | 0.35 | 0.14 |
| Benign motor - cog/aut. vs poor motor - cog/aut | -5.97 | 0.008 | 0.003 |
|  |  |  |  |
| **Method-4 (covariables: age, sex, disease duration, LEDD)** |  |  |  |
| Benign motor vs benign motor - cog. | 1.60 | 0.52 | 4.97 |
| Benign motor vs poor motor - cog. | -3.72 | 0.09 | 0.02 |
| Benign motor - cog. vs poor motor - cog. | -5.32 | 0.06 | 0.005 |

IL-16:

| Group | B | P value | Exp(B) |
| --- | --- | --- | --- |
| **Method-1 (covariables: sex and LEDD)** |  |  |  |
| Young onset vs non-tremor dominant | 0.00 | 0.72 | 1.00 |
| Young onset vs tremor dominant | 0.001 | 0.33 | 1.001 |
| Young onset vs rapid progression | 0.000 | 0.83 | 1.00 |
| Non-tremor dominant vs tremor dominant | 0.001 | 0.45 | 1.001 |
| Non-tremor dominant vs rapid progression | 0.00 | 0.91 | 1.00 |
| Tremor dominant vs rapid progression | -0.001 | 0.39 | 1.00 |
|  |  |  |  |
| **Method-2 (covariables: sex, LEDD)** |  |  |  |
| Young onset - slow prog. vs late onset - fast prog. | -0.001 | 0.62 | 1.00 |
|  |  |  |  |
| **Method-3 (covariables: age, sex, disease duration, LEDD)** |  |  |  |
| Benign motor vs benign motor - cog/aut. | -0.001 | 0.48 | 1.00 |
| Benign motor vs poor motor - cog/aut. | -0.001 | 0.16 | 1.00 |
| Benign motor - cog/aut. vs poor motor - cog/aut | -0.001 | 0.61 | 1.00 |
|  |  |  |  |
| **Method-4 (covariables: age, sex, disease duration, LEDD)** |  |  |  |
| Benign motor vs benign motor - cog. | -0.001 | 0.43 | 1.00 |
| Benign motor vs poor motor - cog. | -0.002 | 0.09 | 1.00 |
| Benign motor - cog. vs poor motor - cog. | -0.001 | 0.59 | 1.00 |

IL-18:

| Group | B | P value | Exp(B) |
| --- | --- | --- | --- |
| **Method-1 (covariables: sex and LEDD)** |  |  |  |
| Young onset vs non-tremor dominant | 0.002 | 0.26 | 1.00 |
| Young onset vs tremor dominant | 0.000 | 0.95 | 1,00 |
| Young onset vs rapid progression | -0.002 | 0.46 | 1.00 |
| Non-tremor dominant vs tremor dominant | -0.002 | 0.35 | 1.00 |
| Non-tremor dominant vs rapid progression | -0.004 | 0.07 | 1.00 |
| Tremor dominant vs rapid progression | -0.002 | 0.40 | 1.00 |
|  |  |  |  |
| **Method-2 (covariables: sex, LEDD)** |  |  |  |
| Young onset - slow prog. vs late onset - fast prog. | -0.003 | 0.26 | 1.00 |
|  |  |  |  |
| **Method-3 (covariables: age, sex, disease duration, LEDD)** |  |  |  |
| Benign motor vs benign motor - cog/aut. | -0.005 | 0.06 | 1.00 |
| Benign motor vs poor motor - cog/aut. | -0.003 | 0.12 | 1.00 |
| Benign motor - cog/aut. vs poor motor - cog/aut | 0.002 | 0.47 | 1,00 |
|  |  |  |  |
| **Method-4 (covariables: age, sex, disease duration, LEDD)** |  |  |  |
| Benign motor vs benign motor - cog. | -0.004 | 0.22 | 1.00 |
| Benign motor vs poor motor - cog. | -0.003 | 0.23 | 1.00 |
| Benign motor - cog. vs poor motor - cog. | 0.001 | 0.72 | 1.00 |

BDNF:

| Group | B | P value | Exp(B) |
| --- | --- | --- | --- |
| **Method-1 (covariables: sex and LEDD)** |  |  |  |
| Young onset vs non-tremor dominant | -0.05 | 0.13 | 0.95 |
| Young onset vs tremor dominant | -0.02 | 0.58 | 0.98 |
| Young onset vs rapid progression | 0.004 | 0.91 | 1.004 |
| Non-tremor dominant vs tremor dominant | 0.03 | 0.42 | 1.03 |
| Non-tremor dominant vs rapid progression | 0.06 | 0.11 | 1.06 |
| Tremor dominant vs rapid progression | 0.03 | 0.45 | 1.03 |
|  |  |  |  |
| **Method-2 (covariables: sex, LEDD)** |  |  |  |
| Young onset - slow prog. vs late onset - fast prog. | 0.02 | 0.57 | 1.02 |
|  |  |  |  |
| **Method-3 (covariables: age, sex, disease duration, LEDD)** |  |  |  |
| Benign motor vs benign motor - cog/aut. | -0.01 | 0.79 | 0.99 |
| Benign motor vs poor motor - cog/aut. | -0.03 | 0.38 | 0.97 |
| Benign motor - cog/aut. vs poor motor - cog/aut | -0.02 | 0.61 | 0.98 |
|  |  |  |  |
| **Method-4 (covariables: age, sex, disease duration, LEDD)** |  |  |  |
| Benign motor vs benign motor - cog. | -0.003 | 0.95 | 1.00 |
| Benign motor vs poor motor - cog. | -0.03 | 0.35 | 0.97 |
| Benign motor - cog. vs poor motor - cog. | -0.03 | 0.52 | 0.97 |

MCP-1:

| Group | B | P value | Exp(B) |
| --- | --- | --- | --- |
| **Method-1 (covariables: sex and LEDD)** |  |  |  |
| Young onset vs non-tremor dominant | -0.001 | 0.43 | 1.00 |
| Young onset vs tremor dominant | -0.001 | 0.76 | 1.00 |
| Young onset vs rapid progression | -0.003 | 0.21 | 1.00 |
| Non-tremor dominant vs tremor dominant | -0.001 | 0.75 | 1.00 |
| Non-tremor dominant vs rapid progression | -0.001 | 0.49 | 1.00 |
| Tremor dominant vs rapid progression | -0.002 | 0.34 | 1.00 |
|  |  |  |  |
| **Method-2 (covariables: sex, LEDD)** |  |  |  |
| Young onset - slow prog. vs late onset - fast prog. | -0.001 | 0.48 | 1.00 |
|  |  |  |  |
| **Method-3 (covariables: age, sex, disease duration, LEDD)** |  |  |  |
| Benign motor vs benign motor - cog/aut. | -0.002 | 0.29 | 1.00 |
| Benign motor vs poor motor - cog/aut. | -0.003 | 0.06 | 1.00 |
| Benign motor - cog/aut. vs poor motor - cog/aut | -0.001 | 0.71 | 1.00 |
|  |  |  |  |
| **Method-4 (covariables: age, sex, disease duration, LEDD)** |  |  |  |
| Benign motor vs benign motor - cog. | -0.002 | 0.34 | 1.00 |
| Benign motor vs poor motor - cog. | -0.003 | 0.08 | 1.00 |
| Benign motor - cog. vs poor motor - cog. | -0.001 | 0.68 | 0.99 |

MDC:

| Group | B | P value | Exp(B) |
| --- | --- | --- | --- |
| **Method-1 (covariables: sex and LEDD)** |  |  |  |
| Young onset vs non-tremor dominant | -0.001 | 0.64 | 1.00 |
| Young onset vs tremor dominant | 0.001 | 0.71 | 1.00 |
| Young onset vs rapid progression | 0.001 | 0.75 | 1.00 |
| Non-tremor dominant vs tremor dominant | 0.002 | 0.39 | 1.00 |
| Non-tremor dominant vs rapid progression | 0.002 | 0.41 | 1.00 |
| Tremor dominant vs rapid progression | 0.000 | 0.93 | 1.00 |
|  |  |  |  |
| **Method-2 (covariables: sex, LEDD)** |  |  |  |
| Young onset - slow prog. vs late onset - fast prog. | 0.000 | 0.99 | 1.00 |
|  |  |  |  |
| **Method-3 (covariables: age, sex, disease duration, LEDD)** |  |  |  |
| Benign motor vs benign motor - cog/aut. | 0.002 | 0.39 | 1.00 |
| Benign motor vs poor motor - cog/aut. | 0.001 | 0.48 | 1.00 |
| Benign motor - cog/aut. vs poor motor - cog/aut | -0.001 | 0.77 | 1.00 |
|  |  |  |  |
| **Method-4 (covariables: age, sex, disease duration, LEDD)** |  |  |  |
| Benign motor vs benign motor - cog. | 0.004 | 0.17 | 1.01 |
| Benign motor vs poor motor - cog. | 0.002 | 0.25 | 1.00 |
| Benign motor - cog. vs poor motor - cog. | -0.001 | 0.62 | 1.00 |

MIP1Beta:

| Group | B | P value | Exp(B) |
| --- | --- | --- | --- |
| **Method-1 (covariables: sex and LEDD)** |  |  |  |
| Young onset vs non-tremor dominant | 0.003 | 0.10 | 1.00 |
| Young onset vs tremor dominant | 0.004 | 0.12 | 1.00 |
| Young onset vs rapid progression | 0.005 | **0.03** | 1.01 |
| Non-tremor dominant vs tremor dominant | 0.000 | 0.81 | 1.00 |
| Non-tremor dominant vs rapid progression | 0.002 | 0.29 | 1.00 |
| Tremor dominant vs rapid progression | 0.001 | 0.48 | 1.00 |
|  |  |  |  |
| **Method-2 (covariables: sex, LEDD)** |  |  |  |
| Young onset - slow prog. vs late onset - fast prog. | 0.003 | 0.08 | 1.00 |
|  |  |  |  |
| **Method-3 (covariables: age, sex, disease duration, LEDD)** |  |  |  |
| Benign motor vs benign motor - cog/aut. | -0.005 | **0.02** | 1.00 |
| Benign motor vs poor motor - cog/aut. | -0.001 | 0.41 | 1.00 |
| Benign motor - cog/aut. vs poor motor - cog/aut | 0.004 | 0.07 | 1.00 |
|  |  |  |  |
| **Method-4 (covariables: age, sex, disease duration, LEDD)** |  |  |  |
| Benign motor vs benign motor - cog. | -0.003 | 0.20 | 1.00 |
| Benign motor vs poor motor - cog. | 0.00 | 0.88 | 1.00 |
| Benign motor - cog. vs poor motor - cog. | 0.004 | 0.18 | 1.00 |

SCF:

| Group | B | P value | Exp(B) |
| --- | --- | --- | --- |
| **Method-1 (covariables: sex and LEDD)** |  |  |  |
| Young onset vs non-tremor dominant | 0.006 | 0.06 | 1.01 |
| Young onset vs tremor dominant | 0.007 | 0.12 | 1.01 |
| Young onset vs rapid progression | 0.002 | 0.67 | 1.00 |
| Non-tremor dominant vs tremor dominant | 0.00 | 0.97 | 1.00 |
| Non-tremor dominant vs rapid progression | -0.005 | 0.18 | 1.00 |
| Tremor dominant vs rapid progression | -0.005 | 0.21 | 1.00 |
|  |  |  |  |
| **Method-2 (covariables: sex, LEDD)** |  |  |  |
| Young onset - slow prog. vs late onset - fast prog. | 0.002 | 0.51 | 1.00 |
|  |  |  |  |
| **Method-3 (covariables: age, sex, disease duration, LEDD)** |  |  |  |
| Benign motor vs benign motor - cog/aut. | 0.006 | 0.09 | 1.01 |
| Benign motor vs poor motor - cog/aut. | 0.000 | 0.98 | 1.00 |
| Benign motor - cog/aut. vs poor motor - cog/aut | -0.006 | 0.05 | 0.99 |
|  |  |  |  |
| **Method-4 (covariables: age, sex, disease duration, LEDD)** |  |  |  |
| Benign motor vs benign motor - cog. | 0.001 | 0.71 | 1.00 |
| Benign motor vs poor motor - cog. | -0.003 | 0.39 | 1.00 |
| Benign motor - cog. vs poor motor - cog. | -0.004 | 0.31 | 1.00 |

TPO:

| Group | B | P value | Exp(B) |
| --- | --- | --- | --- |
| **Method-1 (covariables: sex and LEDD)** |  |  |  |
| Young onset vs non-tremor dominant | -0.07 | 0.92 | 0.94 |
| Young onset vs tremor dominant | 0.52 | 0.49 | 1.67 |
| Young onset vs rapid progression | -0.05 | 0.95 | 0.95 |
| Non-tremor dominant vs tremor dominant | 0.58 | 0.40 | 1.79 |
| Non-tremor dominant vs rapid progression | 0.02 | 0.98 | 1.02 |
| Tremor dominant vs rapid progression | -0.56 | 0.42 | 0.57 |
|  |  |  |  |
| **Method-2 (covariables: sex, LEDD)** |  |  |  |
| Young onset - slow prog. vs late onset - fast prog. | 0.49 | 0.49 | 1.63 |
|  |  |  |  |
| **Method-3 (covariables: age, sex, disease duration, LEDD)** |  |  |  |
| Benign motor vs benign motor - cog/aut. | -0.78 | 0.29 | 0.55 |
| Benign motor vs poor motor - cog/aut. | -1.26 | **0.047** | 0.28 |
| Benign motor - cog/aut. vs poor motor - cog/aut | -0.48 | 0.47 | 0.62 |
|  |  |  |  |
| **Method-4 (covariables: age, sex, disease duration, LEDD)** |  |  |  |
| Benign motor vs benign motor - cog. | -0.75 | 0.36 | 0.47 |
| Benign motor vs poor motor - cog. | -0.89 | 0.15 | 0.41 |
| Benign motor - cog. vs poor motor - cog. | -0.14 | 0.86 | 0.87 |

ENA 78:

| Group | B | P value | Exp(B) |
| --- | --- | --- | --- |
| **Method-1 (covariables: sex and LEDD)** |  |  |  |
| Young onset vs non-tremor dominant | 0.15 | 0.52 | 1.16 |
| Young onset vs tremor dominant | -0.19 | 0.60 | 0.83 |
| Young onset vs rapid progression | 0.15 | 0.60 | 1.16 |
| Non-tremor dominant vs tremor dominant | -0.33 | 0.33 | 0.72 |
| Non-tremor dominant vs rapid progression | 0.01 | 0.98 | 1.01 |
| Tremor dominant vs rapid progression | 0.34 | 0.34 | 1.41 |
|  |  |  |  |
| **Method-2 (covariables: sex, LEDD)** |  |  |  |
| Young onset - slow prog. vs late onset - fast prog. | 0.21 | 0.46 | 1.23 |
|  |  |  |  |
| **Method-3 (covariables: age, sex, disease duration, LEDD)** |  |  |  |
| Benign motor vs benign motor - cog/aut. | 0.14 | 0.54 | 1.16 |
| Benign motor vs poor motor - cog/aut. | -0.16 | 0.49 | 0.86 |
| Benign motor - cog/aut. vs poor motor - cog/aut | -0.30 | 0.18 | 0.74 |
|  |  |  |  |
| **Method-4 (covariables: age, sex, disease duration, LEDD)** |  |  |  |
| Benign motor vs benign motor - cog. | 0.35 | 0.17 | 1.42 |
| Benign motor vs poor motor - cog. | -0.76 | 0.74 | 0.93 |
| Benign motor - cog. vs poor motor - cog. | -0.43 | 0.11 | 0.65 |

GM-CSF:

| Group | B | P value | Exp(B) |
| --- | --- | --- | --- |
| **Method-1 (covariables: sex and LEDD)** |  |  |  |
| Young onset vs non-tremor dominant | 0.01 | 0.80 | 1.01 |
| Young onset vs tremor dominant | 0.04 | 0.51 | 1.04 |
| Young onset vs rapid progression | - | - | - |
| Non-tremor dominant vs tremor dominant | 1.42 | 0.61 | 1.03 |
| Non-tremor dominant vs rapid progression | - | - | - |
| Tremor dominant vs rapid progression |  |  |  |
|  |  |  |  |
| **Method-2 (covariables: sex, LEDD)** |  |  |  |
| Young onset - slow prog. vs late onset - fast prog. | -0.08 | 0.34 | 0.93 |
|  |  |  |  |
| **Method-3 (covariables: age, sex, disease duration, LEDD)** |  |  |  |
| Benign motor vs benign motor - cog/aut. | -0.03 | 0.60 | 0.98 |
| Benign motor vs poor motor - cog/aut. | -0.03 | 0.43 | 0.97 |
| Benign motor - cog/aut. vs poor motor - cog/aut | -0.004 | 0.94 | 1.00 |
|  |  |  |  |
| **Method-4 (covariables: age, sex, disease duration, LEDD)** |  |  |  |
| Benign motor vs benign motor - cog. | - | - | - |
| Benign motor vs poor motor - cog. | -0.06 | 0.33 | 0.94 |
| Benign motor - cog. vs poor motor - cog. | - | - | - |

Table 4: Detailed results of logistic regression analyses for Toronto.

TNF-α:

| Group | B | P value | Exp(B) |
| --- | --- | --- | --- |
| **Method-1 (covariables: sex and LEDD)** |  |  |  |
| Young onset vs non-tremor dominant | -0.04 | 0.14 | 0.97 |
| Young onset vs tremor dominant | 0.02 | 0.51 | 1.02 |
| Young onset vs rapid progression | -0.02 | 0.60 | 0.99 |
| Non-tremor dominant vs tremor dominant | 0.05 | **0.04** | 1.05 |
| Non-tremor dominant vs rapid progression | 0.02 | 0.49 | 1.02 |
| Tremor dominant vs rapid progression | -0.03 | 0.30 | 0.97 |
|  |  |  |  |
| **Method-2 (covariables: sex, LEDD)** |  |  |  |
| Young onset - slow prog. vs late onset - fast prog. | -0.05 | 0.10 | 0.95 |
|  |  |  |  |
| **Method-3 (covariables: age, sex, disease duration, LEDD)** |  |  |  |
| Benign motor vs benign motor - cog/aut. | 0.02 | 0.46 | 1.02 |
| Benign motor vs poor motor - cog/aut. | 0.007 | 0.80 | 1.007 |
| Benign motor - cog/aut. vs poor motor - cog/aut | -0.01 | 0.65 | 0.99 |
|  |  |  |  |
| **Method-4 (covariables: age, sex, disease duration, LEDD)** |  |  |  |
| Benign motor vs benign motor - cog. | 0.02 | 0.45 | 1.02 |
| Benign motor vs poor motor - cog. | -0.01 | 0.68 | 0.99 |
| Benign motor - cog. vs poor motor - cog. | -0.03 | 0.31 | 0.97 |

TNF-β**:**

| Group | B | P value | Exp(B) |
| --- | --- | --- | --- |
| **Method-1 (covariables: sex and LEDD)** |  |  |  |
| Young onset vs non-tremor dominant | 0.01 | 0.30 | 1.01 |
| Young onset vs tremor dominant | 0.01 | **0.045** | 1.01 |
| Young onset vs rapid progression | 0.01 | **0.03** | 1.01 |
| Non-tremor dominant vs tremor dominant | 0.04 | 0.30 | 1.004 |
| Non-tremor dominant vs rapid progression | 0.01 | 0.12 | 1.01 |
| Tremor dominant vs rapid progression | 0.004 | 0.45 | 1.004 |
|  |  |  |  |
| **Method-2 (covariables: sex, LEDD)** |  |  |  |
| Young onset - slow prog. vs late onset - fast prog. | 0.01 | **0.04** | 1.01 |
|  |  |  |  |
| **Method-3 (covariables: age, sex, disease duration, LEDD)** |  |  |  |
| Benign motor vs benign motor - cog/aut. | 0.01 | 0.23 | 1.01 |
| Benign motor vs poor motor - cog/aut. | 0.01 | 0.46 | 1.01 |
| Benign motor - cog/aut. vs poor motor - cog/aut | -0.002 | 0.76 | 1.00 |
|  |  |  |  |
| **Method-4 (covariables: age, sex, disease duration, LEDD)** |  |  |  |
| Benign motor vs benign motor - cog. | -0.001 | 0.78 | 1.00 |
| Benign motor vs poor motor - cog. | 0,004 | 0,37 | 1,004 |
| Benign motor - cog. vs poor motor - cog. | 0,005 | 0,33 | 1,005 |

IL-1alpha:

| Group | B | P value | Exp(B) |
| --- | --- | --- | --- |
| **Method-1 (covariables: sex and LEDD)** |  |  |  |
| Young onset vs non-tremor dominant | -0.23 | 0.73 | 0.80 |
| Young onset vs tremor dominant | -0.02 | 0.97 | 0.98 |
| Young onset vs rapid progression | 1.37 | 0.16 | 3.94 |
| Non-tremor dominant vs tremor dominant | 0.21 | 0.76 | 1.23 |
| Non-tremor dominant vs rapid progression | 1.60 | 0.09 | 4.95 |
| Tremor dominant vs rapid progression | 1.39 | 0.15 | 4.03 |
|  |  |  |  |
| **Method-2 (covariables: sex, LEDD)** |  |  |  |
| Young onset - slow prog. vs late onset - fast prog. | 0.58 | 0.38 | 1.79 |
|  |  |  |  |
| **Method-3 (covariables: age, sex, disease duration, LEDD)** |  |  |  |
| Benign motor vs benign motor - cog/aut. | 0.65 | 0.36 | 1.92 |
| Benign motor vs poor motor - cog/aut. | 1.56 | 0.10 | 4.47 |
| Benign motor - cog/aut. vs poor motor - cog/aut | 0.91 | 0.31 | 2.48 |
|  |  |  |  |
| **Method-4 (covariables: age, sex, disease duration, LEDD)** |  |  |  |
| Benign motor vs benign motor - cog. | 1.10 | 0.15 | 3.01 |
| Benign motor vs poor motor - cog. | -0.22 | 0.74 | 0.80 |
| Benign motor - cog. vs poor motor - cog. | -1.32 | 0.12 | 0.27 |

IL-3:

| Group | B | P value | Exp(B) |
| --- | --- | --- | --- |
| **Method-1 (covariables: sex and LEDD)** |  |  |  |
| Young onset vs non-tremor dominant | 0.63 | 0.43 | 1.88 |
| Young onset vs tremor dominant | -0.20 | 0.81 | 0.82 |
| Young onset vs rapid progression | 1.32 | 0.18 | 3.75 |
| Non-tremor dominant vs tremor dominant | -0.83 | 0.33 | 0.44 |
| Non-tremor dominant vs rapid progression | 0.69 | 0.45 | 2.00 |
| Tremor dominant vs rapid progression | 1.52 | 0.14 | 4.58 |
|  |  |  |  |
| **Method-2 (covariables: sex, LEDD)** |  |  |  |
| Young onset - slow prog. vs late onset - fast prog. | 0.69 | 0.41 | 1.99 |
|  |  |  |  |
| **Method-3 (covariables: age, sex, disease duration, LEDD)** |  |  |  |
| Benign motor vs benign motor - cog/aut. | -1.28 | 0.24 | 0.28 |
| Benign motor vs poor motor - cog/aut. | 0.69 | 0.55 | 2.00 |
| Benign motor - cog/aut. vs poor motor - cog/aut | 1.97 | 0.14 | 7.16 |
|  |  |  |  |
| **Method-4 (covariables: age, sex, disease duration, LEDD)** |  |  |  |
| Benign motor vs benign motor - cog. | -0.72 | 0.44 | 0.49 |
| Benign motor vs poor motor - cog. | -1.54 | 0.18 | 0.22 |
| Benign motor - cog. vs poor motor - cog. | -0.82 | 0.54 | 0.44 |

IL-4:

| Group | B | P value | Exp(B) |
| --- | --- | --- | --- |
| **Method-1 (covariables: sex and LEDD)** |  |  |  |
| Young onset vs non-tremor dominant | -0.09 | 0.60 | 0.92 |
| Young onset vs tremor dominant | 0.05 | 0.69 | 1.06 |
| Young onset vs rapid progression | 0.04 | 0.82 | 1.04 |
| Non-tremor dominant vs tremor dominant | 0.14 | 0.37 | 1.15 |
| Non-tremor dominant vs rapid progression | 0.13 | 0.50 | 1.13 |
| Tremor dominant vs rapid progression | -0.01 | 0.94 | 0.99 |
|  |  |  |  |
| **Method-2 (covariables: sex, LEDD)** |  |  |  |
| Young onset - slow prog. vs late onset - fast prog. | 0.12 | 0.38 | 1.12 |
|  |  |  |  |
| **Method-3 (covariables: age, sex, disease duration, LEDD)** |  |  |  |
| Benign motor vs benign motor - cog/aut. | 0.38 | 0.83 | 1.04 |
| Benign motor vs poor motor - cog/aut. | 0.10 | 0.63 | 1.10 |
| Benign motor - cog/aut. vs poor motor - cog/aut | 0.07 | 0.74 | 1.07 |
|  |  |  |  |
| **Method-4 (covariables: age, sex, disease duration, LEDD)** |  |  |  |
| Benign motor vs benign motor - cog. | -0.18 | 0.38 | 0.84 |
| Benign motor vs poor motor - cog. | -0.23 | 0.23 | 0.79 |
| Benign motor - cog. vs poor motor - cog. | -0.05 | 0.84 | 1.00 |

IL-5:

| Group | B | P value | Exp(B) |
| --- | --- | --- | --- |
| **Method-1 (covariables: sex and LEDD)** |  |  |  |
| Young onset vs non-tremor dominant | -0.53 | 0.70 | 0.59 |
| Young onset vs tremor dominant | -0.50 | 0.73 | 0.63 |
| Young onset vs rapid progression | 0.56 | 0.70 | 1.76 |
| Non-tremor dominant vs tremor dominant | 0.07 | 0.96 | 1.07 |
| Non-tremor dominant vs rapid progression | 1.09 | 0.47 | 2.98 |
| Tremor dominant vs rapid progression | 1.02 | 0.51 | 2.78 |
|  |  |  |  |
| **Method-2 (covariables: sex, LEDD)** |  |  |  |
| Young onset - slow prog. vs late onset - fast prog. | -20.9 | 1.00 | 1.00 |
|  |  |  |  |
| **Method-3 (covariables: age, sex, disease duration, LEDD)** |  |  |  |
| Benign motor vs benign motor - cog/aut. | - | - | - |
| Benign motor vs poor motor - cog/aut. | -0.50 | 0.81 | 0.61 |
| Benign motor - cog/aut. vs poor motor - cog/aut | - | - | - |
|  |  |  |  |
| **Method-4 (covariables: age, sex, disease duration, LEDD)** |  |  |  |
| Benign motor vs benign motor - cog. | - | - | - |
| Benign motor vs poor motor - cog. | 1.15 | 0.44 | 3.17 |
| Benign motor - cog. vs poor motor - cog. | - | - | - |

IL-7:

| Group | B | P value | Exp(B) |
| --- | --- | --- | --- |
| **Method-1 (covariables: sex and LEDD)** |  |  |  |
| Young onset vs non-tremor dominant | -0.01 | 0.80 | 0,99 |
| Young onset vs tremor dominant | -0,01 | 0.84 | 0,99 |
| Young onset vs rapid progression | -0.04 | 0.45 | 0.96 |
| Non-tremor dominant vs tremor dominant | 0.003 | 0.95 | 1.003 |
| Non-tremor dominant vs rapid progression | -0.03 | 0.56 | 0.97 |
| Tremor dominant vs rapid progression | -0.03 | 0.53 | 0.96 |
|  |  |  |  |
| **Method-2 (covariables: sex, LEDD)** |  |  |  |
| Young onset - slow prog. vs late onset - fast prog. | 0.01 | 0.68 | 1.01 |
|  |  |  |  |
| **Method-3 (covariables: age, sex, disease duration, LEDD)** |  |  |  |
| Benign motor vs benign motor - cog/aut. | -0.002 | 0.97 | 0.99 |
| Benign motor vs poor motor - cog/aut. | 0.01 | 0.78 | 1.01 |
| Benign motor - cog/aut. vs poor motor - cog/aut | 0.01 | 0.75 | 1.02 |
|  |  |  |  |
| **Method-4 (covariables: age, sex, disease duration, LEDD)** |  |  |  |
| Benign motor vs benign motor - cog. | -0.02 | 0.75 | 0.99 |
| Benign motor vs poor motor - cog. | -0.11 | **0.04** | 0.89 |
| Benign motor - cog. vs poor motor - cog. | -0.09 | 0.12 | 0.91 |

IL-8:

| Group | B | P value | Exp(B) |
| --- | --- | --- | --- |
| **Method-1 (covariables: sex and LEDD)** |  |  |  |
| Young onset vs non-tremor dominant | -0.04 | 0.58 | 0.96 |
| Young onset vs tremor dominant | -0.01 | 0.85 | 0.99 |
| Young onset vs rapid progression | -0.36 | **0.03** | 0.70 |
| Non-tremor dominant vs tremor dominant | 0.03 | 0.71 | 1.03 |
| Non-tremor dominant vs rapid progression | -0.32 | 0.05 | 0.73 |
| Tremor dominant vs rapid progression | -0.35 | **0.03** | 0.70 |
|  |  |  |  |
| **Method-2 (covariables: sex, LEDD)** |  |  |  |
| Young onset - slow prog. vs late onset - fast prog. | 0.35 | 0.61 | 1.41 |
|  |  |  |  |
| **Method-3 (covariables: age, sex, disease duration, LEDD)** |  |  |  |
| Benign motor vs benign motor - cog/aut. | -0.12 | 0.29 | 0.88 |
| Benign motor vs poor motor - cog/aut. | -0.08 | 0.47 | 0.93 |
| Benign motor - cog/aut. vs poor motor - cog/aut | 0.05 | 0.68 | 1.04 |
|  |  |  |  |
| **Method-4 (covariables: age, sex, disease duration, LEDD)** |  |  |  |
| Benign motor vs benign motor - cog. | -0.16 | 0.09 | 0.85 |
| Benign motor vs poor motor - cog. | -0.08 | 0.33 | 0.92 |
| Benign motor - cog. vs poor motor - cog. | 0.08 | 0.43 | 1.09 |

IL-10:

| Group | B | P value | Exp(B) |
| --- | --- | --- | --- |
| **Method-1 (covariables: sex and LEDD)** |  |  |  |
| Young onset vs non-tremor dominant | -0.29 | 0.18 | 0.74 |
| Young onset vs tremor dominant | -0.14 | 0.49 | 0.87 |
| Young onset vs rapid progression | -0.31 | 0.30 | 0.74 |
| Non-tremor dominant vs tremor dominant | 0.16 | 0.47 | 1.20 |
| Non-tremor dominant vs rapid progression | -0.01 | 0.90 | 0.99 |
| Tremor dominant vs rapid progression | -0.17 | 0.57 | 0.84 |
|  |  |  |  |
| **Method-2 (covariables: sex, LEDD)** |  |  |  |
| Young onset - slow prog. vs late onset - fast prog. | 0.06 | 0.40 | 1.07 |
|  |  |  |  |
| **Method-3 (covariables: age, sex, disease duration, LEDD)** |  |  |  |
| Benign motor vs benign motor - cog/aut. | 0.14 | 0.50 | 1.15 |
| Benign motor vs poor motor - cog/aut. | 0.22 | 0.42 | 1.25 |
| Benign motor - cog/aut. vs poor motor - cog/aut | 0.08 | 0.77 | 1.08 |
|  |  |  |  |
| **Method-4 (covariables: age, sex, disease duration, LEDD)** |  |  |  |
| Benign motor vs benign motor - cog. | 0.13 | 0.53 | 1.14 |
| Benign motor vs poor motor - cog. | -0.17 | 0.42 | 0.85 |
| Benign motor - cog. vs poor motor - cog. | -0,30 | 0.22 | 0.74 |

IL-12p40:

| Group | B | P value | Exp(B) |
| --- | --- | --- | --- |
| **Method-1 (covariables: sex and LEDD)** |  |  |  |
| Young onset vs non-tremor dominant | 0.66 | 0.33 | 1.93 |
| Young onset vs tremor dominant | -0.02 | 0.98 | 0.99 |
| Young onset vs rapid progression | 1.75 | 0.07 | 5.73 |
| Non-tremor dominant vs tremor dominant | -0.67 | 0.32 | 0.51 |
| Non-tremor dominant vs rapid progression | 1.09 | 0.25 | 2.98 |
| Tremor dominant vs rapid progression | 1.76 | 0.07 | 5.82 |
|  |  |  |  |
| **Method-2 (covariables: sex, LEDD)** |  |  |  |
| Young onset - slow prog. vs late onset - fast prog. | 0.75 | 0.72 | 2.10 |
|  |  |  |  |
| **Method-3 (covariables: age, sex, disease duration, LEDD)** |  |  |  |
| Benign motor vs benign motor - cog/aut. | 0.61 | 0.39 | 1.84 |
| Benign motor vs poor motor - cog/aut. | 1.01 | 0.26 | 2.74 |
| Benign motor - cog/aut. vs poor motor - cog/aut | 0.40 | 0.64 | 1.49 |
|  |  |  |  |
| **Method-4 (covariables: age, sex, disease duration, LEDD)** |  |  |  |
| Benign motor vs benign motor - cog. | 0.13 | 0.86 | 1.14 |
| Benign motor vs poor motor - cog. | -2.16 | **0.02** | 0.12 |
| Benign motor - cog. vs poor motor - cog. | -2.28 | **0.03** | 0.10 |

IL-13:

| Group | B | P value | Exp(B) |
| --- | --- | --- | --- |
| **Method-1 (covariables: sex and LEDD)** |  |  |  |
| Young onset vs non-tremor dominant | -0.06 | 0.68 | 0.95 |
| Young onset vs tremor dominant | -0.30 | 0.64 | 0.74 |
| Young onset vs rapid progression | -0.07 | 0.72 | 0.94 |
| Non-tremor dominant vs tremor dominant | -0.25 | 0.15 | 0.78 |
| Non-tremor dominant vs rapid progression | -0.01 | 0.96 | 0.99 |
| Tremor dominant vs rapid progression | 0.24 | 0.26 | 1.27 |
|  |  |  |  |
| **Method-2 (covariables: sex, LEDD)** |  |  |  |
| Young onset - slow prog. vs late onset - fast prog. | 0.03 | 0.84 | 1.03 |
|  |  |  |  |
| **Method-3 (covariables: age, sex, disease duration, LEDD)** |  |  |  |
| Benign motor vs benign motor - cog/aut. | -0.03 | 0.86 | 0.98 |
| Benign motor vs poor motor - cog/aut. | -0.09 | 0.65 | 0.90 |
| Benign motor - cog/aut. vs poor motor - cog/aut | -0.01 | 0.73 | 0.94 |
|  |  |  |  |
| **Method-4 (covariables: age, sex, disease duration, LEDD)** |  |  |  |
| Benign motor vs benign motor - cog. | 0.20 | 0.18 | 1.23 |
| Benign motor vs poor motor - cog. | -0.63 | **0.03** | 0.53 |
| Benign motor - cog. vs poor motor - cog. | -0.84 | **0.007** | 0.43 |

IL-15:

| Group | B | P value | Exp(B) |
| --- | --- | --- | --- |
| **Method-1 (covariables: sex and LEDD)** |  |  |  |
| Young onset vs non-tremor dominant | -2.48 | 0.40 | 0.08 |
| Young onset vs tremor dominant | -3.50 | 0.24 | 0.03 |
| Young onset vs rapid progression | -2.90 | 0.47 | 0.05 |
| Non-tremor dominant vs tremor dominant | -1.02 | 0.75 | 0.36 |
| Non-tremor dominant vs rapid progression | -0.44 | 0.91 | 0.64 |
| Tremor dominant vs rapid progression | 0.58 | 0.89 | 1.78 |
|  |  |  |  |
| **Method-2 (covariables: sex, LEDD)** |  |  |  |
| Young onset - slow prog. vs late onset - fast prog. | 2.40 | 0.35 | 11.2 |
|  |  |  |  |
| **Method-3 (covariables: age, sex, disease duration, LEDD)** |  |  |  |
| Benign motor vs benign motor - cog/aut. | 3.10 | 0.36 | 22.4 |
| Benign motor vs poor motor - cog/aut. | 5.80 | 0.19 | 333.8 |
| Benign motor - cog/aut. vs poor motor - cog/aut | 2.70 | 0.48 | 14.9 |
|  |  |  |  |
| **Method-4 (covariables: age, sex, disease duration, LEDD)** |  |  |  |
| Benign motor vs benign motor - cog. | -0.29 | 0.93 | 0.75 |
| Benign motor vs poor motor - cog. | -0.96 | 0.38 | 0.05 |
| Benign motor - cog. vs poor motor - cog. | -2.68 | 0.50 | 0.07 |

IL-16:

| Group | B | P value | Exp(B) |
| --- | --- | --- | --- |
| **Method-1 (covariables: sex and LEDD)** |  |  |  |
| Young onset vs non-tremor dominant | -0.002 | 0.49 | 0.99 |
| Young onset vs tremor dominant | -0.003 | 0.28 | 0.99 |
| Young onset vs rapid progression | -0.003 | 0.45 | 0.99 |
| Non-tremor dominant vs tremor dominant | -0.001 | 0.67 | 0.99 |
| Non-tremor dominant vs rapid progression | -0.001 | 0.78 | 0.99 |
| Tremor dominant vs rapid progression | 0,00 | 0.96 | 1.0 |
|  |  |  |  |
| **Method-2 (covariables: sex, LEDD)** |  |  |  |
| Young onset - slow prog. vs late onset - fast prog. | -0.003 | 0.29 | 0.99 |
|  |  |  |  |
| **Method-3 (covariables: age, sex, disease duration, LEDD)** |  |  |  |
| Benign motor vs benign motor - cog/aut. | 0,00 | 0.96 | 1.00 |
| Benign motor vs poor motor - cog/aut. | -0.01 | 0.19 | 0.99 |
| Benign motor - cog/aut. vs poor motor - cog/aut | -0.01 | 0.18 | 0.99 |
|  |  |  |  |
| **Method-4 (covariables: age, sex, disease duration, LEDD)** |  |  |  |
| Benign motor vs benign motor - cog. | -0.001 | 0.58 | 0.99 |
| Benign motor vs poor motor - cog. | -0.004 | 0.19 | 0.99 |
| Benign motor - cog. vs poor motor - cog. | -0.02 | 0.46 | 0.99 |

IL-18:

| Group | B | P value | Exp(B) |
| --- | --- | --- | --- |
| **Method-1 (covariables: sex and LEDD)** |  |  |  |
| Young onset vs non-tremor dominant | -0.002 | 0.47 | 0.99 |
| Young onset vs tremor dominant | 0.001 | 0.54 | 1.001 |
| Young onset vs rapid progression | -0.007 | 0.17 | 0.99 |
| Non-tremor dominant vs tremor dominant | 0.003 | 0.21 | 1.003 |
| Non-tremor dominant vs rapid progression | -0.005 | 0.33 | 0.99 |
| Tremor dominant vs rapid progression | -0.008 | 0.10 | 0.99 |
|  |  |  |  |
| **Method-2 (covariables: sex, LEDD)** |  |  |  |
| Young onset - slow prog. vs late onset - fast prog. | -0.004 | 0.15 | 0.99 |
|  |  |  |  |
| **Method-3 (covariables: age, sex, disease duration, LEDD)** |  |  |  |
| Benign motor vs benign motor - cog/aut. | -0.002 | 0.53 | 0.99 |
| Benign motor vs poor motor - cog/aut. | -0.002 | 0.66 | 0.99 |
| Benign motor - cog/aut. vs poor motor - cog/aut | 0.00 | 0.95 | 1.00 |
|  |  |  |  |
| **Method-4 (covariables: age, sex, disease duration, LEDD)** |  |  |  |
| Benign motor vs benign motor - cog. | 0.001 | 0.67 | 1.001 |
| Benign motor vs poor motor - cog. | 0.001 | 0.79 | 1.001 |
| Benign motor - cog. vs poor motor - cog. | 0.00 | 0.87 | 1.00 |

BDNF:

| Group | B | P value | Exp(B) |
| --- | --- | --- | --- |
| **Method-1 (covariables: sex and LEDD)** |  |  |  |
| Young onset vs non-tremor dominant | -0.18 | **0.003** | 0.84 |
| Young onset vs tremor dominant | -0.14 | **0.02** | 0.87 |
| Young onset vs rapid progression | -0.06 | 0.40 | 0.94 |
| Non-tremor dominant vs tremor dominant | 0.04 | 0.41 | 1.04 |
| Non-tremor dominant vs rapid progression | 0.12 | 0.09 | 1.12 |
| Tremor dominant vs rapid progression | 0.07 | 0.27 | 1.08 |
|  |  |  |  |
| **Method-2 (covariables: sex, LEDD)** |  |  |  |
| Young onset - slow prog. vs late onset - fast prog. | -0.04 | 0.38 | 0.96 |
|  |  |  |  |
| **Method-3 (covariables: age, sex, disease duration, LEDD)** |  |  |  |
| Benign motor vs benign motor - cog/aut. | -0.03 | 0.55 | 0.97 |
| Benign motor vs poor motor - cog/aut. | -0.04 | 0.59 | 0.96 |
| Benign motor - cog/aut. vs poor motor - cog/aut | -0.01 | 0.91 | 0.99 |
|  |  |  |  |
| **Method-4 (covariables: age, sex, disease duration, LEDD)** |  |  |  |
| Benign motor vs benign motor - cog. | 0.04 | 0.40 | 1.04 |
| Benign motor vs poor motor - cog. | 0.01 | 0.76 | 1.01 |
| Benign motor - cog. vs poor motor - cog. | -0.02 | 0.63 | 0.98 |

MCP-1:

| Group | B | P value | Exp(B) |
| --- | --- | --- | --- |
| **Method-1 (covariables: sex and LEDD)** |  |  |  |
| Young onset vs non-tremor dominant | 0.00 | 0.92 | 1.00 |
| Young onset vs tremor dominant | 0.00 | 0.97 | 1.00 |
| Young onset vs rapid progression | -0.003 | 0.41 | 1.00 |
| Non-tremor dominant vs tremor dominant | 0.00 | 0.95 | 1.00 |
| Non-tremor dominant vs rapid progression | -0.003 | 0.36 | 1.00 |
| Tremor dominant vs rapid progression | -0.003 | 0.39 | 1.00 |
|  |  |  |  |
| **Method-2 (covariables: sex, LEDD)** |  |  |  |
| Young onset - slow prog. vs late onset - fast prog. | -0.001 | 0.61 | 0.99 |
|  |  |  |  |
| **Method-3 (covariables: age, sex, disease duration, LEDD)** |  |  |  |
| Benign motor vs benign motor - cog/aut. | 0.00 | 0.98 | 1.00 |
| Benign motor vs poor motor - cog/aut. | 0.001 | 0.84 | 1.00 |
| Benign motor - cog/aut. vs poor motor - cog/aut | 0.001 | 0.81 | 1.00 |
|  |  |  |  |
| **Method-4 (covariables: age, sex, disease duration, LEDD)** |  |  |  |
| Benign motor vs benign motor - cog. | 0.001 | 0.61 | 1.00 |
| Benign motor vs poor motor - cog. | 0.00 | 0.99 | 1.00 |
| Benign motor - cog. vs poor motor - cog. | -0.001 | 0.66 | 0.99 |

MDC:

| Group | B | P value | Exp(B) |
| --- | --- | --- | --- |
| **Method-1 (covariables: sex and LEDD)** |  |  |  |
| Young onset vs non-tremor dominant | 0.00 | 0.90 | 1.00 |
| Young onset vs tremor dominant | 0.00 | 0.97 | 1.00 |
| Young onset vs rapid progression | -0.003 | 0.41 | 0.99 |
| Non-tremor dominant vs tremor dominant | 0.00 | 0.95 | 1.00 |
| Non-tremor dominant vs rapid progression | -0.003 | 0.36 | 0.99 |
| Tremor dominant vs rapid progression | -0.003 | 0.39 | 0.99 |
|  |  |  |  |
| **Method-2 (covariables: sex, LEDD)** |  |  |  |
| Young onset - slow prog. vs late onset - fast prog. | -0.001 | 0.61 | 0.99 |
|  |  |  |  |
| **Method-3 (covariables: age, sex, disease duration, LEDD)** |  |  |  |
| Benign motor vs benign motor - cog/aut. | 0.00 | 0.98 | 1.00 |
| Benign motor vs poor motor - cog/aut. | 0.001 | 0.84 | 1.00 |
| Benign motor - cog/aut. vs poor motor - cog/aut | 0.001 | 0.81 | 1.00 |
|  |  |  |  |
| **Method-4 (covariables: age, sex, disease duration, LEDD)** |  |  |  |
| Benign motor vs benign motor - cog. | 0.001 | 0.61 | 1.00 |
| Benign motor vs poor motor - cog. | 0.00 | 0.99 | 1.00 |
| Benign motor - cog. vs poor motor - cog. | -0.001 | 0.66 | 0.99 |

MIP1Beta:

| Group | B | P value | Exp(B) |
| --- | --- | --- | --- |
| **Method-1 (covariables: sex and LEDD)** |  |  |  |
| Young onset vs non-tremor dominant | -0.004 | 0.18 | 0,99 |
| Young onset vs tremor dominant | -0.003 | 0.32 | 0.99 |
| Young onset vs rapid progression | -0.001 | 0.70 | 0.99 |
| Non-tremor dominant vs tremor dominant | 0.001 | 0.70 | 1.00 |
| Non-tremor dominant vs rapid progression | 0.002 | 0.49 | 1.00 |
| Tremor dominant vs rapid progression | 0.001 | 0.70 | 1.00 |
|  |  |  |  |
| **Method-2 (covariables: sex, LEDD)** |  |  |  |
| Young onset - slow prog. vs late onset - fast prog. | 0.00 | 0.94 | 1,00 |
|  |  |  |  |
| **Method-3 (covariables: age, sex, disease duration, LEDD)** |  |  |  |
| Benign motor vs benign motor - cog/aut. | -0.01 | 0.11 | 0.99 |
| Benign motor vs poor motor - cog/aut. | -0.001 | 0.87 | 0.99 |
| Benign motor - cog/aut. vs poor motor - cog/aut | 0.004 | 0.23 | 1.00 |
|  |  |  |  |
| **Method-4 (covariables: age, sex, disease duration, LEDD)** |  |  |  |
| Benign motor vs benign motor - cog. | 0.00 | 0.89 | 1.00 |
| Benign motor vs poor motor - cog. | 0.001 | 0.80 | 1.00 |
| Benign motor - cog. vs poor motor - cog. | 0.001 | 0.74 | 1.00 |

SCF:

| Group | B | P value | Exp(B) |
| --- | --- | --- | --- |
| **Method-1 (covariables: sex and LEDD)** |  |  |  |
| Young onset vs non-tremor dominant | -0.001 | 0.82 | 0.99 |
| Young onset vs tremor dominant | -0.005 | 0.20 | 0.99 |
| Young onset vs rapid progression | -0.002 | 0.68 | 0.99 |
| Non-tremor dominant vs tremor dominant | -0.004 | 0.30 | 0.99 |
| Non-tremor dominant vs rapid progression | -0.001 | 0.80 | 0.99 |
| Tremor dominant vs rapid progression | 0.003 | 0.57 | 1.00 |
|  |  |  |  |
| **Method-2 (covariables: sex, LEDD)** |  |  |  |
| Young onset - slow prog. vs late onset - fast prog. | -0.002 | 0.50 | 0.99 |
|  |  |  |  |
| **Method-3 (covariables: age, sex, disease duration, LEDD)** |  |  |  |
| Benign motor vs benign motor - cog/aut. | -0.003 | 0.37 | 0.99 |
| Benign motor vs poor motor - cog/aut. | -0.001 | 0.83 | 0.99 |
| Benign motor - cog/aut. vs poor motor - cog/aut | 0.002 | 0.54 | 1.00 |
|  |  |  |  |
| **Method-4 (covariables: age, sex, disease duration, LEDD)** |  |  |  |
| Benign motor vs benign motor - cog. | 0.001 | 0.79 | 1.00 |
| Benign motor vs poor motor - cog. | 0.00 | 0.93 | 1.00 |
| Benign motor - cog. vs poor motor - cog. | -0.001 | 0.76 | 0.99 |

TPO:

| Group | B | P value | Exp(B) |
| --- | --- | --- | --- |
| **Method-1 (covariables: sex and LEDD)** |  |  |  |
| Young onset vs non-tremor dominant | -0.16 | 0.22 | 0.85 |
| Young onset vs tremor dominant | -0.18 | 0.14 | 0.83 |
| Young onset vs rapid progression | 0.13 | 0.27 | 1.14 |
| Non-tremor dominant vs tremor dominant | -0.03 | 0.85 | 0.97 |
| Non-tremor dominant vs rapid progression | 0.29 | 0.06 | 1.34 |
| Tremor dominant vs rapid progression | 0.32 | **0.04** | 1.37 |
|  |  |  |  |
| **Method-2 (covariables: sex, LEDD)** |  |  |  |
| Young onset - slow prog. vs late onset - fast prog. | 0.02 | 0.86 | 1.02 |
|  |  |  |  |
| **Method-3 (covariables: age, sex, disease duration, LEDD)** |  |  |  |
| Benign motor vs benign motor - cog/aut. | -0.02 | 0.87 | 0.98 |
| Benign motor vs poor motor - cog/aut. | 0.07 | 0.61 | 1.07 |
| Benign motor - cog/aut. vs poor motor - cog/aut | 0.09 | 0.49 | 1.10 |
|  |  |  |  |
| **Method-4 (covariables: age, sex, disease duration, LEDD)** |  |  |  |
| Benign motor vs benign motor - cog. | 0.03 | 0.80 | 1.03 |
| Benign motor vs poor motor - cog. | -0.02 | 0.88 | 0.98 |
| Benign motor - cog. vs poor motor - cog. | -0.05 | 0.73 | 0.96 |

ENA 78:

| Group | B | P value | Exp(B) |
| --- | --- | --- | --- |
| **Method-1 (covariables: sex and LEDD)** |  |  |  |
| Young onset vs non-tremor dominant | -0.06 | 0.74 | 0.94 |
| Young onset vs tremor dominant | -0.06 | 0.76 | 0.94 |
| Young onset vs rapid progression | 0.14 | 0.41 | 1.20 |
| Non-tremor dominant vs tremor dominant | 0,001 | 1.0 | 1.00 |
| Non-tremor dominant vs rapid progression | 0.20 | 0.23 | 1.22 |
| Tremor dominant vs rapid progression | 0.20 | 0.27 | 1.20 |
|  |  |  |  |
| **Method-2 (covariables: sex, LEDD)** |  |  |  |
| Young onset - slow prog. vs late onset - fast prog. | 0.16 | 0.39 | 1.17 |
|  |  |  |  |
| **Method-3 (covariables: age, sex, disease duration, LEDD)** |  |  |  |
| Benign motor vs benign motor - cog/aut. | -0.37 | 0.30 | 0.69 |
| Benign motor vs poor motor - cog/aut. | 0.03 | 0.85 | 1.00 |
| Benign motor - cog/aut. vs poor motor - cog/aut | 0.4 | 0.27 | 1.50 |
|  |  |  |  |
| **Method-4 (covariables: age, sex, disease duration, LEDD)** |  |  |  |
| Benign motor vs benign motor - cog. | 0.01 | 0.94 | 1.01 |
| Benign motor vs poor motor - cog. | 0.24 | 0.12 | 1.27 |
| Benign motor - cog. vs poor motor - cog. | 0.22 | 0.18 | 1.25 |

Table 5: Results of [18F] FEPPA-PET imaging of brain regions between patients with low and high IL-12p40 values with independent samples t-test.

|  | Low IL-12p40 group (n=9)  mean (SD) | High IL-12p40 group (n=9)  mean (SD) | P value |
| --- | --- | --- | --- |
| Frontal | 11.9 (5.0) | 14.5 (4.7) | 0.27 |
| Temporal | 10.3 (4.2) | 12.5 (4.0) | 0.28 |
| Occipital | 11.2 (4.3) | 14.4 (5.3) | 0.19 |
| Thalamus | 11.5 (5.5) | 13.7 (4.7) | 0.39 |
| Striatum | 7.7 (3.7) | 8.9 (3.2) | 0.50 |
| Cingulate | 8.7 (3.5) | 9.6 (3.4) | 0.61 |
| Insula | 9.6 (3.4) | 11.1 (3.8) | 0.40 |
| Cerebellum | 9.3 (3.6) | 11.1 (3.9) | 0.37 |

[18F] FEPPA-PET values depict the total distribution volume (V_T_), of the radioligand concentration.

**References**

[1] Goetz CG, Stebbins GT, Tilley BC (2012) Calibration of unified Parkinson’s disease rating scale scores to *Movement* Disorder Society-unified Parkinson’s disease rating scale scores. *Mov. Disord.* **27**, 1239–1242.
